# Supplementary material for: Katdetectr: an R/bioconductor package utilizing unsupervised changepoint analysis for robust kataegis detection
Source: Gigascience. 2023 Oct 17;12:giad081. doi: 10.1093/gigascience/giad081 (PMC10580377; doi:10.1093/gigascience/giad081)
Supplement: giad081_GIGA-D-23-00051_Revision_1 [file giad081_giga-d-23-00051_revision_1.pdf]

GigaScience

Katdetectr: An R/Bioconductor package utilizing unsupervised changepoint analysis for robust kataegis detection

--Manuscript Draft--

**Manuscript Number:**	GIGA-D-23-00051R1
**Full Title:**	Katdetectr: An R/Bioconductor package utilizing unsupervised changepoint analysis for robust kataegis detection
**Article Type:**	Technical Note
**Funding Information:**	Daniel den Hoed Fonds (DDHF-CCBC) — Dr Harmen van de Werken
**Abstract:**	Background<br>Kataegis refers to the occurrence of regional genomic hypermutation in cancer and is a phenomenon that has been observed in a wide range of malignancies. A kataegis locus constitutes a genomic region with a high mutation rate, i.e., a higher frequency of closely interspersed somatic variants than the overall mutational background. It has been shown that kataegis is of biological significance and possibly clinically relevant. Therefore, an accurate and robust workflow for kataegis detection is paramount.<br>Findings<br>Here we present Katdetectr, an open-source R/Bioconductor-based package for the robust yet flexible and fast detection of kataegis loci in genomic data. In addition, Katdetectr houses functionalities to characterize and visualize kataegis and provides results in a standardized format useful for subsequent analysis. In brief, Katdetectr imports industry-standard formats (MAF, VCF, and VRanges), determines the intermutation distance of the genomic variants and performs unsupervised changepoint analysis utilizing the Pruned Exact Linear Time search algorithm followed by kataegis calling according to user-defined parameters.<br>We used synthetic data and an a priori labeled pan-cancer dataset of whole genome sequenced malignancies for the performance evaluation of Katdetectr and five publicly available kataegis detection packages. Our performance evaluation shows that Katdetectr is robust regarding tumor mutational burden (TMB) and shows the fastest mean computation time. Additionally, Katdetectr reveals the highest accuracy (0.99, 0.99) and normalized Matthews Correlation Coefficient (0.98, 0.92) of all evaluated tools for both datasets.<br>Conclusions<br>Katdetectr is a robust workflow for the detection, characterization, and visualization of kataegis and is available on<br>Bioconductor: https://doi.org/doi:10.18129/B9.bioc.katdetectr
**Corresponding Author:**	Harmen van de Werken<br>Erasmus MC<br>Rotterdam, Select State NETHERLANDS
**Corresponding Author Secondary Information:**	
**Corresponding Author's Institution:**	Erasmus MC
**Corresponding Author's Secondary Institution:**	
**First Author:**	Daan Hazelaar
**First Author Secondary Information:**	
**Order of Authors:**	Daan Hazelaar
	Job van Riet
	Youri Hoogstrate
	Harmen van de Werken
**Order of Authors Secondary Information:**	

Powered by Editorial Manager® and ProduXion Manager® from Aries Systems Corporation

Response to Reviewers:	Please note that we have added a supplementary pdf file that contains our response to the editor and the reviewers. This supplementary files contains mathematical expressions which we use in our response to the reviewers.<br><br>Concerning: GIGA-D-23-00051 and detailed response to its review<br><br>Dear Hongling Zhou,<br><br>Thank you very much for the thorough evaluation of our manuscript GIGA-D-23-00051 entitled: Katdetectr: An R/Bioconductor package utilizing unsupervised changepoint analysis for robust kataegis detection" by Daan Hazelaar; Job van Riet; Youri Hoogstrate; Harmen van de Werken.<br><br>We greatly appreciate the opportunity to revise our manuscript according to the high-quality reports of the reviewers. We include a point-by-point reply to the criticism and suggestions by the reviewers and you. Moreover, the described changes are indicated with track changes in the resubmitted manuscript.<br><br>1. Register any new software application in the bio.tools and SciCrunch.org databases to receive RRID (Research Resource Identification Initiative ID) and biotoolsID identifiers, and include these in your manuscript.<br><br>Dear dr. Hongling Zhou, we have registered katdetectr on bio.tools (biotoolsID: katdetectr)) and SciCrunch.org (RRID: SCR_023506) and added the accompanying identifiers to the manuscript under the section: Availability and requirements in compliance with the GIGA journal requirements.<br><br>2. Computational workflows should be registered in workflowhub.eu and the DOIs cited in the relevant places in the manuscript.<br><br>We have registered katdetectr (10.48546/workflowhub.workflow.463.1) and the performance evaluation of katdetectr (10.48546/workflowhub.workflow.500.1) on workflowhub.eu and added the corresponding to the availability and requirements section in the manuscript.<br><br>Sincerely yours,<br><br>Harmen J. G. van de Werken, Ph.D.<br>Assistant Professor in Computational Biology & Bioinformatics in Immunology and Cancer at the Erasmus Medical Center in the Department of Immunology<br><br>Reviewer #1: minor revision<br>In this short manuscript, Hazelaar et al. describe a new software package written in R, called "katdetectr". This package can be useful as an addition to existing computational tools for identifying and characterizing kataegis in cancer genomes. The paper then compares katdetectr favorably against other software for detecting kataegis, using synthetic and real cancer data. Overall, the paper is fine and the katdetectr package is a nice addition for researchers' toolbox. I would suggest that the authors make the following improve-ments.<br><br>1. Choose a convention for decimal point and digit separator, then stick with it. "." was

used as both the decimal point and digit separator for large numbers, which gets confusing. Typically, "." Is used for decimal point and "," is used for digit separator.

We thank the reviewer for this editorial comment. We have indeed revised our manuscript (and figures) in accordance the convention of using "." as a decimal point and using "," as a digit separator. We apologize for the previous oversight.

2. The Introduction is so abbreviated that it doesn't serve much purpose. Either flesh it out with more information or just drop it completely. This journal accepts papers that go right into re-sults, so it's fine. But the authors should also consider if writing a more expansive introduction can make the paper more accessible to readers who aren't already as knowledgeable.

According to the reviewer's suggestion, we have extended the introduction to improve this manu-script's accessibility for readers not yet familiar with kataegis. Additionally, we have included additional references within the introduction to promote further reading into the current state of re-search regarding kataegis (lines 60 - 78).

3. The biggest issue is using the 2013 Alexandrov kataegis calls as "ground truth" when multi-ple packages published since then detect 102 loci that Alexandrov (2013) missed. Seems like it would be much more sensible to use the calls from the 2020 PCAWG paper instead: https://doi.org/10.1038/s41586-020-1969-6. The data are controlled access, but it should be possible to get them.

Whilst we agree with the reviewer that utilizing the latest release of the kataegis calls (as called within the PCAWG) would be a worthwhile endeavor as the PCAWG-calls would indeed be more recent and potentially contain improved annotations. However, this dataset is currently (as mentioned) only available under controlled-access whilst the Alexandrov et al. call-set is publicly available.

In line with the philosophy of open science and Giga Science, we believe that reproducible and continued benchmarking of novel computational methodology against comparable methodology is paramount and that this is restricted when controlled-access data in involved.

Therefore, we used the publicly-available dataset as described by Alexandrov et al. (2013) for benchmarking which allowed us to co-publish our input data and results for public review and future comparison without restriction.

To overcome the potential inaccuracy of the employed ground-truth call-set, we compared the evaluated methodologies without the dependence of the "ground-truth" labels by employing a Venn diagram (Fig. 2b) which highlights the (shared) dis/concordance against the "ground-truth". This allows for a visual comparison of the packages which is less dependent on the input.

We have extended and refined our discussion to address these valid concerns on the employed "ground-truth" set (lines 287 - 289).

4. Katdetectr does outperform other packages for high TMB samples (≧10). But those are rela-tively few (< 10% of samples). Should state this clearly in text.

We have added the number of currently-investigated WGS samples with a TMB≧ 10 (n = 20) to our manuscript (line 186).

The large pan-cancer analysis by Priestley et al. (2019)[1] on metastatic cancers revealed that 17.7% of examined malignancies reveal TMB ≧ 10 and that this is not a rare occurrence for several malignancies. In particular, metastatic skin and lung

Powered by Editorial Manager® and ProduXion Manager® from Aries Systems Corporation

malignancies reveal 55-60% cases with such elevated TMB. We have further elaborated these potential use-cases within our discussion (line 260 - 261).

[1] Priestley P, Baber J, Lolkema MP, et al. Pan-cancer whole-genome analyses of metastatic solid tumours. Nature. 2019;575(7781):210-216. doi:10.1038/s41586-019-1689-y

5. The runtime data would be better represented by violin plots. Having many data points bunched together isn't helpful to visualize the distributions.

As per reviewer request, we have replaced the boxplots in fig. 2C and suppl. fig. 2 with violin plots and individual data-points.

6. I tested the katdetectr package and noticed something peculiar about the documentation. In section 6 "More parameter settings", there's a disclaimer that the developers did not test such settings. Doesn't seem like a good practice to put that in there if the devs themselves don't know how the function will behave.

We thank the reviewer for extensively investigating katdetectr and commenting on the accompanying vignette.

We would like to emphasize that we have thoroughly tested all available functions presented within katdetectr (incl. unit-testing) to ensure future sanity and proper function. In addition, katdetectr adheres to the BioConductor guidelines and follows their formal programmatic style, testing and documentation.

We merely wished to highlight additional functionality of the presented methodology and the flexibility of the user-available parameters by showcasing an additional use-case involving clustered mutations which do not necessarily adhere to the canonical kataegis ruleset. Whilst we ensured that these additional results were sane, we did not perform an extensive evaluation and comparison of these additional functionalities similar to those we performed for the detection of kataegis.

We agree with the reviewer that this could be misconstrued and derailing from the main functionality of katdetectr, as evaluated within this manuscript, and have removed this section from the vignette.

We updated katdetectr on BioConductor, but please note that the Bioconductor release branch is only updated twice per year (incl. the change in the vignette). The most current version of katdetectr which already includes this change is available from GitHub: https://github.com/ErasmusMC-CCBC/katdetectr

Reviewer #2: major revision

This manuscript presents a clever tool of hypermutation detection with changepoint analysis-based R languages, katdetectr. The authors have constructed the R package based on the changepoint package of Killick and Eckley.

1. In the mutation processing step, the author stated that "the imported variants are pre-processed such that, per chromosome, all variants are sorted in ascending order based on their genomic position. Overlapping variants are merged into a single record." What does "all variants" refer to?

With all variants, we referred to all the genomic variants as supplied by the user within their VCF, MAF or user-curated VRanges object. Users can perform pre-filtering of

genomic variants by utilizing the VRanges object (e.g., as generated from a VCF) and supplying this VRanges into the kataegis detection method. This VRanges can house SNVs, (long) InDels and structural variants and all will be used for downstream kataegis detection if present.

We apologize for the previous omission of details and have extended this within our manuscript (line 358 - 359).

2. Are other variants, e.g., long Indel and structure variation included?

As also mentioned in the previous comment (#1), all forms of genomic variants can be supplied to katdetectr and used for subsequent kataegis detection. The presented analysis and evaluation of kataegis calls as presented within this manuscript was performed on SNVs-only as at least one package only imported SNVs.

Katdetectr merges (partially) overlapping genomic variants (regions) using IRanges::reduce() and from this generates a single record with the 5'-most shared position as reference anchor (start position), an X as reference allele, XX as alternative allele and containing information detailing which variant records were merged.

However, it would be advisable to filter all or large (e.g., >1kb) structural variations beforehand as these could potentially overlap with many (smaller) genomic variants resulting in a potential loss of kataegis detection.

We have extended our methodology with these details on merging overlapping variants (line 358 - 360)

3. How do the other tools deal with such variants, and what's your consideration for this treatment?

To better address this interesting question, we performed an investigation on how the alternative packages handle (long) and overlapping variants as the respective papers, manuscripts, vignettes and manuals lack much (if any) detail on this topic.

We added an additional script to our public repository which we used to assess the behavior of these packages regarding overlapping variants:
https://github.com/ErasmusMC-CCBC/evaluation_katdetectr/blob/main/notebooks/R/checking_overlapping_variants.Rmd

With this script, we generated a small synthetic sample-set of (non-)overlapping variants:
1 InDel (1200 kb)
10 random SNVs
10 kataegis SNVs
9 SNVs that overlap with one or more of the previous
10 SNVs at exactly the same genomic location

If no merging is performed, 40 variants should be present in the resulting data-tables. If merging is performed (such as in katdetectr), only 22 variants should be present. This allows us to empirically determine the (default) behavior of the packages as the documentation and respective application notes are scarce on details regarding overlapping variants.

Please see below, comment #4, for the details of this analysis. In summary, none of the (other) evaluated packages performed merging of overlapping variants.

Maftools
We did not find relevant parameters regarding overlapping variants and no reference to overlapping variants in the documentation, paper or manual of this tools.

From our test-set, we observed that the overlapping variants are not merged.

SeqKat
We did not find relevant parameters regarding overlapping variants and no reference to overlapping variants in the documentation, paper or manual of this tools. SeqKat furthermore only allows the import of a BED file containing SNVs, disregarding anything larger than 1bp.

From our remaining test-set of SNVs-only, we observed that the overlapping variants are not merged.

ClusteredMutations
We did not find relevant parameters regarding overlapping variants and no reference to overlapping variants in the documentation, paper or manual of this tools.

From our test-set, we observed that the overlapping variants are not merged.

SigProfilerClusters
We did not find relevant parameters regarding overlapping variants and no reference to overlapping variants in the documentation, paper or manual of this tools.

From our test-set, we observed that the overlapping variants are not merged.

Kataegis
We did not find relevant parameters regarding overlapping variants and no reference to overlapping variants in the documentation, paper or manual of this tools.

From our test-set, we observed that the overlapping variants are not merged.

4. What are "overlapping variants"?

Please see comment #3 for an explanation of the algorithm and internal handling. Please see the supplementary rebuttal pdf file that contains mathematical expressions which we use to respond to this important question.

5. Why should they be merged?

We deemed merging overlapping genomic variants necessary as we currently do not implement phasing of alleles or include clonal cancer fractions for detection of kataegis to ease the interpretation and accessibility of katdetectr for a general audience. Therefore, if two overlapping variants would not be merged, they would contain a negative or 0 IMD. This could inflate the detection of kataegis whilst likely reflecting an admixture of clones with mutations on alternate genomes / haplotypes or an altogether complex genomic rearrangement. (line 360)

Please note that any merged records will always contain unique metadata ("revmap") detailing the merged variants, a reference allele of X and an alternative allele of XX. This allows user to manually further investigate these regions.

6. Are there any outcomes of these treatments here?

Within all 1024 synthetic samples constituting a total of 21,299,360 SNVs, 4592 SNVs (0.02%) were merged to a single datapoint.

Within all 507 evaluated WGS samples (Alexandrov et al. 2013) constituting a total of 3,382,751 SNV, no SNVs were merged which likely reflects a pre-filtering step within the initial dataset by the authors.

Powered by Editorial Manager® and ProduXion Manager® from Aries Systems Corporation

7. There is a lookup table for chromosome length of UCSC hg19 (in function_performChangepointDetection.R). Does this tool also support other reference genomes of different species or different versions of human genomes? If so, how can users change this parameter?

The previous version (v1.0.0) as deposited at submission of this article indeed only (erroneously) contained a lookup table for hg19. We previously addressed this reviewer's concern in the following git issue: https://github.com/ErasmusMC-CCBC/katdetectr/issues/1

On 26-04-2023, the release branch of BioConductor was updated which includes this update (katdetectr v1.2.0). This updated version of katdetectr contains the argument "refSeq" in detectKataegis() which can be used to specify which human reference genome (by supplying "hg19" or "hg38") should be considered. Additionally, this argument can be used to supply the necessary sequence length for analysis other genomes; allowing for the analysis of additional organisms.

We have also included additional information within the vignette detailing this, please see section: "Analyzing non-standard sequences" in the vignette accompanied with the katdetectr package (v1.2.0):
https://bioconductor.org/packages/devel/bioc/vignettes/katdetectr/inst/doc/General_overview.html

8. The authors tested four algorithms of changepoint package for kataegis detection, and found the PELT algorithm outperformed the others. The authors have described the results roughly, could the authors state the reasons in mathematical aspect more detailly? And are these methods recommended in another scenario?

Whilst this is an interesting question, we feel that Killick and Eckley[1,2] have already expertly detailed the various mathematical intricacies of these algorithms, as employed within the changepoint package. These excellent works contain the information concerning; mathematical proofs, computational complexity, definitions of the search algorithms, possible loss functions and their implications, methods for guarding against overfitting, changes in mean, changes in variance, changes in mean and variance, and more examples.

Within our manuscript, we opted to forego this introduction to focus on the empirical performance of these search methods in the context of kataegis detection within WGS data.

[1] R. Killick, P. Fearnhead & I. A. Eckley (2012) Optimal Detection of Changepoints With a Linear Computational Cost, Journal of the American Statistical Association, 107:500, 1590-1598, DOI: 10.1080/01621459.2012.737745

[2] Killick, R., & Eckley, I. A. (2014). changepoint: An R Package for Changepoint Analysis. Journal of Statistical Software, 58(3), 1–19. https://doi.org/10.18637/jss.v058.i03

9. I noticed you have added one pseudo IMD in the distance from the last variant to the end of the DNA sequence to make the rates detection in change point analysis equal the mutation rate of the entire chromosome. Why this process is necessary?

Please see the supplementary rebuttal pdf file that contains mathematical expressions which we use to respond to this relevant question.

10. Except for these four algorithms, do you have any plan for implementing other algorithms for this package?

To our understanding, PELT is the current state-of-the-art search algorithm for changepoint analysis. Therefore, have currently employed this as the default algorithm. Nevertheless, we implemented katdetectr in a flexible and open-source manner which allows us or other contributors to easily implement additional search methods when requested. As PELT provided us with overall good results regarding kataegis detection, we do not foresee the immediate usage of alternate methods.

11. In the performance evaluation, you have the same variants files tested with different tools with default parameters. As we know, the tools with PCF algorithms may have parameters of penalty for each discontinuity in the curve. What are these parameters set default in these tools?

Both MafTools and Kataegis mostly employ a Piecewise Constant Fit (PCF) methodology for kataegis detection. To the best of our knowledge, we did not discern a relevant parameter in maftools (maftools::rainfallPlot()) which concerns the "penalty for each discontinuity" therefore we cannot comment on this further.

Within the package Kataegis, the kataegis::kata() function contains the "gamma" parameter for which the manual states that this sets the "penalty for each discontinuity in the curve" and is by default set to 25 (and was also left default during our performance evaluation).

We have sought to perform all alternative tools utilizing their hard-coded or otherwise suggested default settings as mentioned by the authors in their respective manuscripts and/or manuals to the best of our ability (line 600 - 618). Katdetectr was likewise performed with its defaults settings as described within our manuscript and/or hard-coded default values.

12. Are there any influences on the kataegis detection?

As also mentioned in comment #11, we have sought to perform all alternative tools utilizing their hard-coded or otherwise suggested default settings as mentioned by the authors in their respective manuscripts and/or manuals to the best of our ability (line 621 - 638). Katdetectr was likewise performed with its defaults settings as described within our manuscript and/or hard-coded default values. We have not performed additional parameter sweeps for the alternative packages as we argue that the default settings will be used by the majority of users. We therefore cannot discard that fine-tuning the parameters would have an influence on the current evaluation.

We have added this limitation to the discussion (line 307 - 312).

13. For different tools you have convert the datasets to different formats, i.e., MAF, BED, why do you choose MAF as the input format and how do you keep the input data consistency in all these different formats?

Within katdetectr, we provide functions to import VCF and MAF files or custom VRanges. However, several other evaluated packages were only capable of importing MAF or BED files. Therefore, we converted the variant data into the preferred formats as specified in the respective manuals of each package. Each time, we checked the consistency of the transformed data to exclude possible artefacts during conversion.

All utilized code for the importing and transformation of the data can be found in our GitHub repository:https://github.com/ErasmusMC-CCBC/evaluation_katdetectr/

14. For the evaluation scores, could the authors provide raw score of true positive and

	true negative other than TPR and TNR?<br><br>Supplementary tables 1 and 2 contain the raw data detailing all true positives, false positives, true negatives, and false negatives per package for the synthetic and WGS datasets respectively.<br><br>15. In addition, the deposited data for performance evaluation is not accessible outside my workplace. And more detailed instructions are necessary for the data. After I loaded the data named parameters_synthetic_data.RData in R, I was lost for deeper looking into the data. When I tried to direct the loaded data to an object, a text of "chr "parameters"" was echoed.<br><br>To ease further reproducibility of our work, we have implemented a Jupyter (R) Notebook in which the various steps of the comparison can be reproduced in a virtual environment (or within a local R environment when installing the IRkernel package): https://github.com/ErasmusMC-CCBC/evaluation_katdetectr/blob/main/notebooks/1.EvaluatePackages.ipynb<br><br>In addition, this notebook contains a code snippet (using zen4R) which can automatically download all our initial input and generated results directly from Zenodo:https://dx.doi.org/10.5281/zenodo.6810477<br><br>These downloaded data can then be used in the downstream visualization and performance evaluations code-blocks. We hope this eases the reviewer reproduction of the initial dataset and following steps leading to the (re-)production of all presented figures and tables.
**Additional Information:**	
**Question**	**Response**
Are you submitting this manuscript to a special series or article collection?	No
**Experimental design and statistics**<br><br>Full details of the experimental design and statistical methods used should be given in the Methods section, as detailed in our Minimum Standards Reporting Checklist. Information essential to interpreting the data presented should be made available in the figure legends.<br><br>Have you included all the information requested in your manuscript?	Yes
**Resources**<br><br>A description of all resources used, including antibodies, cell lines, animals and software tools, with enough information to allow them to be uniquely	Yes

identified, should be included in the Methods section. Authors are strongly encouraged to cite Research Resource Identifiers (RRIDs) for antibodies, model organisms and tools, where possible.<br><br>Have you included the information requested as detailed in our Minimum Standards Reporting Checklist?	
**Availability of data and materials**<br><br>All datasets and code on which the conclusions of the paper rely must be either included in your submission or deposited in publicly available repositories (where available and ethically appropriate), referencing such data using a unique identifier in the references and in the "Availability of Data and Materials" section of your manuscript.<br><br>Have you have met the above requirement as detailed in our Minimum Standards Reporting Checklist?	Yes

Manuscript

Click here to access/download;Manuscript;main_text_katdetectr_without_tra

Katdetectr: An R/Bioconductor package utilizing unsupervised changepoint analysis for robust kataegis detection

Daan M. Hazelaar[1, †] d.hazelaar@erasmusmc.nl

Job van Riet[1-2, †, ^] job.vanriet@dkfz-heidelberg.de

Youri Hoogstrate[3] y.hoogstrate@erasmusmc.nl

Harmen J. G. van de Werken[2,4, *] h.vandewerken@erasmusmc.nl

[1]Department of Medical Oncology, Erasmus MC Cancer Institute, Erasmus University Medical Center, Dr. Molewaterplein 40, 3015 GD, Rotterdam, the Netherlands.

[2]Department of Urology, Erasmus MC Cancer Institute, Erasmus University Medical Center, Dr. Molewaterplein 40, 3015 GD, Rotterdam, the Netherlands.

[3]Department of Neurology, Erasmus MC Cancer Institute, Erasmus University Medical Center, Dr. Molewaterplein 40, 3015 GD, Rotterdam, the Netherlands,

[4]Department of Immunology, Erasmus MC Cancer Institute, Erasmus University Medical Center, Dr. Molewaterplein 40, 3015 GD, Rotterdam, the Netherlands.

[^]Current Address: Division of AI in Oncology, German Cancer Research Center (DKFZ), Im Neuenheimer Feld 280, 69120, Heidelberg, Germany

[†]Shared first-authorship

[*]Corresponding author

ORCID iDs: Daan Mattijn Hazelaar [0000-0002-7513-6813]; Job van Riet [0000-0001-7767-7923]; Youri Hoogstrate [0000-0003-2166-0676]; Harmen van de Werken [0000-0002-9794-1477];

Abstract

Background

Kataegis refers to the occurrence of regional genomic hypermutation in cancer and is a phenomenon that has been observed in a wide range of malignancies. A kataegis locus constitutes a genomic region with a high mutation rate, i.e., a higher frequency of closely interspersed somatic variants than the overall mutational background. It has been shown that kataegis is of biological significance and possibly clinically relevant. Therefore, an accurate and robust workflow for kataegis detection is paramount.

Findings

Here we present *Katdetectr,* an open-source R/Bioconductor-based package for the robust yet flexible and fast detection of kataegis loci in genomic data. In addition, *Katdetectr* houses functionalities to characterize and visualize kataegis and provides results in a standardized format useful for subsequent analysis. In brief, *Katdetectr* imports industry-standard formats (MAF, VCF, and VRanges), determines the intermutation distance of the genomic variants and performs unsupervised changepoint analysis utilizing the Pruned Exact Linear Time search algorithm followed by kataegis calling according to user-defined parameters.

We used synthetic data and an *a priori* labeled pan-cancer dataset of Whole Genome Sequenced malignancies for the performance evaluation of Katdetectr and five publicly available kataegis detection packages. Our performance evaluation shows that Katdetectr is robust regarding tumor mutational burden (TMB) and shows the fastest mean computation time. Additionally, Katdetectr reveals the highest accuracy (0.99, 0.99) and normalized Matthews Correlation Coefficient (0.98, 0.92) of all evaluated tools for both datasets.

Conclusions

Katdetectr is a robust workflow for the detection, characterization, and visualization of kataegis and is available on Bioconductor: https://doi.org/doi:10.18129/B9.bioc.katdetectr

Keywords: Kataegis, R-package**,** Bioconductor, Changepoint analysis, Cancer.

Introduction

Large-scale next-generation sequencing of malignancies has revealed that a myriad of mutational mechanisms and mutational rates are at play within even a single tumor genome. Moreover, it has been shown that mutations can cluster together, i.e., the acquired mutations are found in proximity to one another, much closer than expected if each base-pair had an equal probability of being mutated. This phenomenon was termed kataegis and its respective genomic location was termed a kataegis locus [1, 2].

Kataegis, Greek for thunderstorm or shower, was first observed and visualized in whole genome sequencing (WGS) data of 21 primary breast cancers [1]. Alexandrov and colleagues, subsequently, detected 873 kataegis loci in a pan-cancer dataset containing 507 WGS samples from primary malignancies [2].

Extensive exploration of the etiology of kataegis revealed a significant positive association between kataegis and two distinct mutational signatures (COSMIC signatures SBS2 and SBS13) both attributed to the APOBEC enzyme-family [3, 4]. Subsequently, multiple studies confirmed the importance of the APOBEC enzymes in cancer, showing that APOBEC enzymes are a major cause of mutagenesis, grouped in clusters, dispersed throughout the cancer genome and in extrachromosomal DNA[5–7]. Additionally, kataegis has been ascribed in lymphomas to two other mutational signatures (COSMIC signatures SBS84 and SBS85) related to the APOBEC family member Activation-induced cytidine deaminase (AID) enzyme [8].

Moreover, the locations of kataegis loci have been associated with locations of somatic structural variant breakpoints. Kataegis loci have been observed most frequently within the proximity of deletions and complex rearrangement breakpoints [3, 9]. Furthermore, kataegis can occur within known cancer driver genes including

TP53, EGFR and *BRAF* which are associated with overall survival in some cancer types [5]. However, the clinical relevance of kataegis remains to be validated and therefore obfuscates kataegis as a clinical biomarker for prognosis. Moreover, future insight into kataegis etiology and clinical applications requires accurate and robust detection of kataegis.

Since the discovery of kataegis, different computational detection tools using genomic variant data have been developed and are publicly available, including; MafTools [10], ClusteredMutations [11], kataegis [12], SeqKat [13] and, SigProfilerClusters [14]. These packages employ distinct statistical methods for kataegis detection and differ in their ease of use and computational feasibility. Therefore, a comparison of their performances is currently needed.

Here, we introduce Katdetectr, an R-based Bioconductor package that contains a suite for the detection, characterization, and visualization of kataegis. Additionally, we have evaluated and compared the performance of Katdetectr to the five commonly used and publicly available kataegis detection packages.

Results

The principle of Katdetectr is to assess the variation in the mutation rate of a cancer genome. To achieve this, Katdetectr starts by importing and preprocessing industry-standard variant calling formats (VCF, MAF, VRanges) (**Figure 1A**). Next, the Intermutation Distance (IMD) is determined, which denotes the distance between variants in base-pairs (**Figure 1B,** see Methods). Unsupervised changepoint analysis is performed, using the IMD as input, which results in detected changepoints. The changepoints, which denote the points at which the distribution of the IMD changes, are used to segment the genomic sequence. Finally, segments are annotated and labeled as a putative kataegis locus if a segment fits the user-defined settings: the mean IMD of the segment ≤ *IMDcutoff* and the number of variants in the segment ≥ *minSizeKataegis*. The IMD, segmentation, and detected kataegis loci can be visualized by Katdetectr in a rainfall plot (**Figure 1C**).

Figure 1, Overview of the Katdetectr workflow, Intermutation distance, and rainfall plots. A) General workflow of Katdetectr from data import to data visualization represented by arrows. B) The intermutation distance (IMD) is determined for all genomic variants in each

chromosome, and rainfall plots are used to visualize the IMDs. Single Nucleotide Variant (SNV), Multi Nucleotide Variant (MNV). C) Rainfall plot of WGS breast cancers sample PD7049a as interrogated by Katdetectr with IMDcutoff = 1,000 and minSizeKataegis = 6 [2]. Y-axis: IMD, x-axis: variant ID ordered on genomic location, light blue rectangles: kataegis loci with genomic variants within kataegis loci shown in bold. The color depicts the mutational type. The vertical lines represent detected changepoints, while black horizontal solid lines show the mean IMD of each segment.

Katdetectr search algorithm selection

To optimize Katdetectr for kataegis detection, we generated a synthetic dataset to test four changepoint search algorithms, namely; Pruned Exact Linear Time (PELT) [15], Binary Segmentation (BinSeg) [15], Segment Neighbourhoods (SegNeigh) [17], and At Most One Change (AMOC). The synthetic dataset contains 1024 samples with a varying number of kataegis loci and Tumor Mutational Burden (TMB) (see Methods). All variants in this dataset were binary labeled for kataegis, as a variant either lies within a kataegis locus (TRUE) or not (FALSE). This dataset was considered ground truth and was used for computing performance metrics. We analyzed the synthetic dataset separately for each search algorithm showing that the PELT algorithm outperformed the alternatives (**Supplementary table 1, supplementary figure 1, 2**). Therefore, we set PELT as the default search algorithm in Katdetectr.

Performance evaluation

We utilized the synthetic dataset to evaluate the performances of Katdetectr and five publicly available kataegis detection packages: MafTools, ClusteredMutations, Kataegis, SeqKat, and, SigProfilerClusters (**Table 1, supplementary table 1**). Katdetectr revealed the highest overall accuracy (0.99), normalized Matthews Correlation Coefficient (nMCC: 0.98), and F1 score (0.97), whereas ClusteredMutations showed the highest True Positive Rate (TPR: 0.99) and Kataegis showed the highest True Negative Rate (TNR: 0.99). Most packages showed a high nMCC for samples with a TMB ranging from 0.1 - 50. However, the performance of all packages dropped for samples with a TMB ≥ 100 (**Figure 2A**). More specifically, for Katdetectr and Kataegis, this is due to an increase in false negatives. For SeqKat, MafTools, ClusteredMutations, and SigProfilerClusters, this performance drop is due to an increase in false positives in samples with a TMB of 100 and 500 (**Supplementary figure 1**).

Next to the synthetic dataset, we evaluated the performance of the kataegis detection packages on a dataset containing 507 *a priori* labeled Whole Genome Sequenced (WGS) samples from Alexandrov *et al.* (see Methods) [2]. Katdetectr revealed the highest overall accuracy (0.99), nMCC (0.92), and F1 score (0.83), whereas ClusteredMutations showed the highest TPR (0.99) and SigProfilerClusters showed the highest TNR (0.99) (**Table 1, Supplementary figure 1)**. Katdetectr, ClusteredMutations, and MafTools showed a high nMCC (>0.92) on the samples with a low or middle TMB. However, the performance of all packages drops for samples with a TMB >10 (n = 20) (**figure 2A**). This is due to an increase in false negatives by Kataegis and SeqKat and false positives by Katdetectr, MafTools, ClusteredMutations, and SigProfilerClusters.

Summary and performance of kataegis detection packages.

					Synthetic dataset					WGS dataset				
Package	Reference	Availible on	Language	Method	Accuracy	nMCC	F1	TPR	TNR	Accuracy	nMCC	F1	TPR	TNR
Katdetectr	Hazelaar, van Riet et al., 2023	Bioconductor	R	Changepoint analysis (PELT)	0.99	0.98	0.97	0.94	0.99	0.99	0.92	0.83	0.91	0.99
SeqKat	Taylor et al., 2013	CRAN	R	Sliding window / exact binomial test	0.84	0.54	0.02	0.93	0.84	0.99	0.85	0.69	0.59	0.99
MafTools	Mayakonda et al., 2018	Bioconductor	R	Sliding window / PCF	0.74	0.53	0.01	0.96	0.74	0.99	0.85	0.66	0.93	0.99
SigProfilerClusters	Bergstrom, Kundu, et al., 2022	Github	Python	Model sample specific IMD cutoff	0.65	0.52	0.01	0.88	0.65	0.99	0.84	0.68	0.66	0.99
ClusteredMutations	Lora, 2016	CRAN	R	Anti-Robinson matrix	0.70	0.53	0.01	0.99	0.74	0.99	0.83	0.61	0.99	0.99
Kataegis	Lin et al., 2021	Github	R	PCF	0.99	0.80	0.52	0.36	0.99	0.99	0.56	0.03	0.02	0.99

Table 1. Summary information of all evaluated kataegis detection packages and their respective performance metrics regarding kataegis classification on 1,024 synthetic samples and 507 a priori labeled Whole Genome Sequenced (WGS) samples. Accuracy, normalized Matthews Correlation Coefficient (nMCC), F1 score, True Positive Rate (TPR) and True Negative Rate (TNR), Pruned Exact Linear Time (PELT), Piecewise Constant Fit (PCF), Intermutation Distance (IMD).

We visualized the concordance regarding per sample kataegis classification and kataegis locus between Katdetectr, SigProfilerClusters, ClusteredMutations, MafTools, and the original authors of the WGS dataset: Alexandrov *et al., 2013* (**Figure 2B**). In total, 451 kataegis loci were detected in 127 WGS samples by all the packages and the original publication. Interestingly, Katdetectr, SigProfilerClusters, ClusteredMutations, and MafTools concordantly detected 102 previously unannotated kataegis loci within the original publication.

The runtimes of all packages were recorded to give insight into the computational feasibility of these packages. Katdetectr showed the lowest mean runtime on both the synthetic and the WGS datasets (**figure 2C**).

Figure 2. Performance evaluation of kataegis detection tools. A) The normalized Matthews Correlation Coefficient (nMCC) per package and Tumor Mutational Burden (TMB) class is depicted by individual data points connected with a dashed line (colored per package). B) Venn diagrams showing the concordance between Katdetectr, SigProfilerClusters, MafTools, ClusteredMutations, and Alexandrov et al.

regarding kataegis classification per sample (i.e., does a sample contain one or more kataegis loci) and per kataegis loci (i.e., does a detected kataegis locus overlap with a kataegis locus detected by another package). C) Boxplots with individual data points represent the per sample runtimes of kataegis detection packages on the synthetic and Whole Genome Sequence datasets. Boxplots were sorted in ascending order based on mean runtime (depicted in the text below the boxplot). Y-axis is log_{10}-scaled. Boxplots depict the Inter Quartile Range, with the median as a black horizontal line.

Katdetectr examples with different TMBs

We highlight four samples from the datasets that illustrate how Katdetectr accurately detects kataegis loci regardless of the TMB of the respective sample (**Figure 3**). The synthetic sample 124625_1_50_100 (TMB: 500) harbors one kataegis locus, containing 57 variants, which is detected by Katdetectr (**Figure 3A**). This kataegis locus is also detected by SeqKat, MafTools, ClusteredMutations, and SigProfilerClusters, in addition to numerous false positives. The package Kataegis did not detect any kataegis loci in this synthetic sample.

In lung adenocarcinoma sample LUAD-E01014 (TMB: 7.6), Katdetectr detected 37 kataegis loci containing 449 variants (**Figure 3B**). MafTools, ClusteredMutations, and SeqKat detected similar kataegis loci in this sample, whereas Kataegis and SigProfilerClusters did not detect any kataegis loci in this sample. In breast cancer sample PD7207a (TMB: 0.8), two kataegis loci were detected by Katdetectr MafTools, ClusteredMutations, and SigProfilerClusters (**Figure 3C**). Kataegis and SeqKat did not detect any kataegis loci in this sample. Lastly, in the breast cancer sample PD4086a (TMB: 0.6), one kataegis locus was detected by all packages except for Kataegis (**Figure 3D**).

Figure 3. Rainfall plots constructed by Katdetectr and confusion matrices, accuracy, and nMCC for four samples. A) Synthetic sample 124625_1_50_100 with Tumor Mutational Burden (TMB): 500, B) Lung adenocarcinoma Whole Genome Sequenced (WGS) sample LUAD-E01014 with TMB: 7.6. C) Breast cancer WGS sample PD7207a with TMB: 2.5. D) Breast cancer WGS sample PD4086a with TMB: 0.62. The WGS samples were collected and labeled for kataegis by Alexandrov *et al.;* their results were used as ground truth to construct the confusion matrices and performance metrics [2]. Rainfall plot: Y-axis: IMD, x-axis: variant ID ordered on genomic location, light blue rectangles: kataegis loci with genomic variants within kataegis loci shown in bold. The color depicts the mutational type. The vertical lines represent detected changepoints, while black horizontal solid lines show the mean IMD of each segment. Confusion matrix: True Positive (TP), False Positive (FP), True Negative (TN), False Negative (FN), Accuracy, and normalized Matthews Correlation Coefficient (nMCC).

Discussion

Here, we described Katdetectr, an R/Bioconductor package for the detection, characterization, and visualization of kataegis in genomic variant data by utilizing unsupervised changepoint analysis.

First, we tested four search algorithms for changepoint analysis, which revealed that the PELT [15] algorithm outperformed the BinSeg [16], SegNeigh [17], and AMOC algorithms both in terms of prediction accuracy and computational feasibility. The BinSeg algorithm performed reasonably well, however, it underfitted the data, which resulted in many false negatives. The SegNeigh algorithm performed well on samples with a TMB < 5; however, this algorithm is computationally expensive, as it scales exponentially with the size of the data, and cannot reasonably be used for the analysis of samples with a TMB > 10. Unsurprisingly, the AMOC (at most one change) algorithm cannot detect kataegis as a kataegis locus is generally defined by two changepoints.

Besides testing search algorithms, we benchmarked Katdetectr using PELT and five publicly available kataegis detection packages which were recently published and used for supporting kataegis research [2, 5, 14, 15]. Since no consensus benchmark was available, we aimed to get insight into the performance of these tools. The complexity of kataegis detection is to separate genomic regions of higher-than-expected mutational density from the background of somatic mutations. Therefore, we argued that generating a synthetic dataset containing samples of varying TMB (0.1-500), would provide a good measure for algorithmic solvability of the kataegis detection problem. Benchmarking on this synthetic dataset revealed that the accuracy of kataegis detection for all evaluated packages drops when the TMB increases. Performance evaluation per TMB-binned class revealed that Katdetectr is on par with alternative packages for samples with low or middle TMB. However, in contrast to alternative packages, Katdetectr remained robust when analyzing samples with a high TMB. This could be an important feature when analyzing late-stage (metastatic) malignancies or malignancies with a known predisposition of acquiring many somatic mutations such as skin or lung malignancies [20]. Additionally, the computation times of Katdetectr are feasible for samples with a TMB ranging from 0.1 to 500 as PELT scales linearly with the size of the data [15]. This shows that kataegis detection using Katdetectr is feasible on reasonably modern computer hardware.

The presented performance evaluation depends on the truth labels provided by the datasets. Both the synthetic and the WGS dataset have their limitations. We constructed the synthetic dataset by modeling mutations on a genome as a Bernoulli process, which is a common approach for modeling events that occur in a sequence. However, we did not incorporate prior biological knowledge in the synthetic dataset generation. Both SeqKat and SigProfilerClusters incorporate biological assumptions regarding kataegis, e.g., mutation context, which possibly negatively influenced their performance regarding the synthetic dataset. Additionally, the distance between events generated by a Bernoulli process is a geometric random variable. For a large n, which is the case for a human genome, a geometric random variable approximates an exponential random variable. Since we constrain Katdetectr to only fit exponential distributions it is unsurprising that Katdetectr performs well on the synthetic dataset. Nevertheless, MafTools, ClusteredMutations, SeqKat, and SigProfilerClusters are less robust when analyzing the synthetic samples with a TMB of 100 and 500 as they classify many false positives kataegis loci.

In addition to the synthetic dataset, we used the *a priori* labeled pan-cancer WGS dataset from the groundbreaking work of Alexandrov *et al.* to evaluate the kataegis detection tools [2]. However, the field of kataegis has grown and evolved since the publication of this dataset. Therefore, we want to emphasize that this dataset should not be considered an unequivocal truth, and the performance metrics should not be taken at face value. The annotation of this dataset likely contains several false positives and false negatives; as highlighted by the concordant discovery of 102 additional kataegis loci by several packages. Nevertheless, we believe that the current benchmarking results give insight into the behavior of the evaluated packages regarding kataegis classification in samples with varying TMB. Additionally, the dataset published by Alexandrov and the predictions by all tools evaluated here are publicly available which facilitates benchmarking of future endeavors regarding kataegis loci detection methods.

Our benchmarking showed that, for the WGS dataset, Katdetectr, MafTools, ClusteredMutations, and, SigProfilerClusters have a high concordance in classifying a whole sample as kataegis positive or negative. However, when concerning distinct kataegis loci, we observed more differences. ClusteredMutations reported the overall largest number of loci (n = 2,360), indicating it has the highest sensitivity. Conversely, kataegis (n = 8) and SeqKat (n = 528) reported the overall smallest number of loci which we deem too small based on visual

inspection. The third smallest number of kataegis loci is reported by SigProfilerClusters (n = 764), indicating it has the highest specificity. Katdetectr appears to balance sensitivity and specificity as it only detects kataegis loci detected by one or more alternative packages (n = 1,050).

We have sought to test the performance of all alternative tools utilizing their hard-coded or otherwise suggested default settings as mentioned by the authors in their respective manuscripts or manuals. Katdetectr was likewise performed with its default settings as described within this manuscript. We have not performed additional parameter sweeps for the alternative packages as we argue that the default settings will be used by the majority of users. We therefore cannot discard that fine-tuning the parameters would have had an influence on our performance evaluation.

Kataegis is the most commonly used term for local hypermutations and has historically been defined as a cluster of at least six variants, of which the mean IMD is less or equal to 1000 base pairs [1, 16]. However, this definition has been altered recently, making the formal definition of kataegis ambiguous [2, 4, 5, 14]. For instance, another type of clustered mutations is called Omikli, which refers to clusters smaller than kataegis, generally containing three or four variants [7]. Although different types of clustered variants can be detected using Katdetectr by supplying the correct parameters, we only evaluated Katdetectr for the detection of kataegis.

We made Katdetectr publicly available on the Bioconductor platform, which requires peer-reviewed open-source software and high standards regarding development, documentation, and unit testing. Furthermore, Bioconductor ensures reliability and operability on common operating systems (Windows, macOS, and Linux). We designed Katdetectr to fit well in the Bioconductor ecosystem by incorporating common Bioconductor object classes. This allows Katdetectr to be used reciprocally with the plethora of statistical software packages available in Bioconductor for preprocessing and subsequent analysis. Lastly, we implemented Katdetectr flexibly, allowing Katdetectr to be used in an ad hoc manner for quick assessment of clustered variants and extensive research of the mutation rates across a tumor genome.

Conclusion

Katdetectr is a free, open-source R package available on Bioconductor that contains a suite for the detection, characterization, and visualization of kataegis. Katdetectr employs the PELT search algorithm for unsupervised changepoint analysis, resulting in robust and fast kataegis detection. Additionally, Katdetectr has been implemented in a flexible manner which allows Katdetectr to expand in the field of kataegis. Katdetectr is available on Bioconductor[21] and on GitHub[22].

Methods

Implementation Katdetectr

Katdetectr (v1.2.0, git commit 5a6e5d04109eb082cbea040049dca34237b6c8f5) was developed in the R statistical programming language (v4.2.0) [23]. Katdetectr imports genomic variants through generic, standardized file formats for variant calling: MAF, VCF, or Bioconductor-standard VRanges objects. Within Katdetectr, the imported variants are pre-processed such that, per chromosome, all variants (all rows in variant file; incl. InDels or structural variations) are sorted in ascending order based on their genomic position. Overlapping variants are merged into a single record as phasing and clonality are not considered by katdetectr. Following, per $chromosome_j$, the intermutation distance ($IMD_{i,j}$) of each $variant_{i,j}$ and its closest upstream $variant_{i-1,j}$ is calculated according to;

$$IMD_{i,j} = \begin{cases} i = 1 & s_{i,j} \\ i > 1 & s_{i,j} - s_{i-1,j} \end{cases} \qquad i = \{1, 2, \ldots, k_j\}$$

Equation 1

With i as the variant number, j as the chromosome number, s as the genomic location of the first base-pair of a $variant_{i,j}$ and k_j as the total number of variants in $chromosome_j$ (**Figure 1B**). Additionally, for each $chromosome_j$ one pseudo IMD, $IMD_{p,j}$, is added such that;

$$n_j = IMD_{p,j} + \sum_{i=1}^{k_j} IMD_{i,j}$$

Equation 2

With n_j as the total number of base-pairs in $chromosome_j$

Katdetectr aims to identify genomic regions characterized by specific mutation rates. An unsupervised technique called changepoint analysis is performed per chromosome on the IMDs to assess the variability in mutation rate across each chromosome. Changepoint analysis refers to the process of detecting points in a sequence of observations where the statistical properties of the sequence significantly change. Subsequently, the detected changepoints are used to segment the input sequence into segments. For a detailed description of the changepoint analysis, see the work of Killick, Fearnhead, and Eckley [15]

We implemented the `cpt.meanvar()` function from the commonly used R changepoint package (v2.2.3) in Katdetectr for the unsupervised segmentation of IMDs, as detailed by [11, 20, 21]. We set the following parameters settings; method: Pruned Exact Linear Time (PELT), minimal segment length: 2, test statistic: Exponential, and penalty: Bayesian Information Criterion (BIC), as default settings in Katdetectr.

After changepoint analysis, each segment is annotated with its respective genomic start and end positions, its mean IMD, and the total number of included variants. Since we use an exponential distribution as the test statistic in changepoint analysis, each segment has a corresponding rate parameter of the fitted exponential distribution. Whereas each segment is annotated with its corresponding mutation rate, the mutation rate of an entire sample can be expressed as the weighted arithmetic mean of the mutation rate of the segments;

$$\lambda_t = \frac{k_t}{n_t} = \sum_{s=1}^{m} \frac{\lambda_s n_s}{n_t}$$

Equation 3

With λ_t as the mutation rate of the entire sample, k_t as the total number of variants present in the sample, n_t as the total number of base pairs in the genome, m as the total number of segments in the sample, and λ_s and n_s as the mutation rate and the number of base-pairs in $segment_s$

To call a segment a putative kataegis locus, it has to adhere to two user-defined parameters: the maximum mean IMD of the segment (*IMDcutoff*) and the minimum number of included variants (*minSizeKataegis*). These

parameters can be provided as static integer values or as a custom R function determining the IMD cutoff for each segment. For example, the following function for annotation of kataegis events, as was used by the ICGC/TCGA Pan-Cancer Analysis of Whole Genomes Consortium, can be easily implemented in Katdetectr [3]:

$$IMDcutoff_s \leq \frac{-log\left(1 - \sqrt[k_s-1]{\frac{0.01}{L_s}}\right)}{\lambda_{med}}$$

$$with;\ \lceil IMDcutoff \rceil = 1000$$

Equation 4

With $IMDcutoff_s$ as the IMD cut-off value, k_s as the number of mutations and L_s as the length of $segment_s$ in base-pairs. For this function the rate of the whole sample is modeled assuming an exponential distribution with;

$$\lambda_{med} = \frac{log(2)}{median(IMD)}$$

Equation 5

Henceforth, all segments satisfying these user-specified parameters are considered putative kataegis loci and stored appropriately. Two or more adjacent kataegis loci are merged and stored as a single record.

The output of Katdetectr consists of an S4 object of class "KatDetect" which stores all relevant information regarding kataegis detection and characterization. A KatDetect object contains four slots: 1) the putative kataegis loci (Granges), 2) the detected segments (Granges), 3) the inputted genomic variants with annotation (Vranges), and 4) the parameters settings (List). These data objects can be accessed using accessor functions.

In addition, we implemented three methods for the KatDetect class, *summary*, *show,* and *rainfallPlot*. In concordance with R standards, the *summary* function prints a synopsis of the performed analysis, including the

number of detected kataegis loci; and the number of variants inside a kataegis loci. The *show* function displays information regarding the S4 class and the synopsis.

The method *rainfallPlot* is a function for generating rainfall plots. These rainfall plots display the genomic ordered IMDs (from all genomic variants) within a sample and highlight putative kataegis loci and associated genomic variants. This function has additional arguments: *showSequence,* which allow the user to display specific chromosomes, and *showSegmentation,* for displaying the changepoints and the mean IMD of all segments.

For additional examples and more hands-on technical instructions, we refer to the accompanying vignette (**Supplemental vignette**) or the online Bioconductor repository[21].

Performance evaluation

As multiple packages for kataegis detection are publicly available, we compared Katdetectr against MafTools (v2.13.0), ClusteredMutations (v1.0.1), kataegis (v0.99.2), SeqKat (v0.0.8) and, SigProfilerClusters (v1.0.11) [6-10]. For benchmarking, we used an in-house generated synthetic dataset and an *a priori* labeled pan-cancer dataset of whole genome sequenced malignancies. As not all evaluated packages accepted InDels

We used the following definition of kataegis as postulated by Alexandrov and colleagues: a kataegis locus is 1) a continuous segment harboring ≥6 variants and 2) the captured IMDs within the segment have a mean IMD of ≤1000 bp [2]. To quantify and compare performances, the task of kataegis detection was reduced to a binary classification problem. The task of the kataegis detection packages was to correctly label each variant for kataegis, i.e., whether or not a genomic variant lies within a kataegis locus.

Performance metrics

Only a small fraction of all observed variants is located within kataegis loci, this results in a large class imbalance which renders the interpretation of performance metrics, such as accuracy, F1, TPR, and TNR, counterintuitive and possibly unrepresentative (**Equation 3**). Therefore, the normalized Matthews Correlation

Coefficient (nMCC) was used as the primary metric for performance evaluation. The nMCC considers performance proportionally to both the size of positive and negative elements in a dataset [26].

$$Accuracy = \frac{TP + TN}{TP + FP + TN + FN}$$

$$MCC = \frac{TP \cdot TN - FP \cdot FN}{\sqrt{(TP + FP) \cdot (TP + FN) \cdot (TN + FP) \cdot (TN + FN)}}$$

$$nMCC = \frac{MCC + 1}{2}$$

$$F1 = \frac{TP}{TP + \frac{1}{2}(FP + FN)}$$

$$TPR = \frac{TP}{TP + FN}$$

$$TNR = \frac{TN}{TN + FP}$$

Equation 6. Performance metrics. Accuracy, Matthews Correlation Coefficient (MCC), normalized Matthews Correlation Coefficient (nMCC), F1 score, True Positive Rate (TPR), and True Negative Rate (TNR).

True Positive (TP): Predicted: variant in kataegis locus. Truth set: variant in kataegis locus.
False Positive (FP): Predicted: variant in kataegis locus. Truth set: variant not in kataegis locus.
True Negative (TN): Predicted: variant not in kataegis locus. Truth set: variant not in kataegis locus.
False Negative (FN): Predicted: variant not in kataegis locus. Truth set: variant in kataegis locus.

We utilized Venn diagrams to display the concordance of the kataegis detection packages. We showed in which samples the packages detected one or more kataegis loci and which kataegis loci were detected by the packages. Two packages are said to detect the same kataegis locus if the genomic locations of their respective kataegis locus overlap by at least one base pair.

To give insight into the package's computation time, the packages runtime performance was recorded using the `proc.time()` function from the base R package. All packages and comparisons were run on the same server utilizing an AMD EPYC 7742 64-Core Processor. The packages Katdetectr and SigProfilerClusters contained options for parallel processing and used at most four cores per sample during the analyses. All other packages used a single processing core per sample.

All scripts necessary for running and visualizing the performance evaluation of all evaluated packages are available on GitHub[22]. All data used for the performance evaluation is available at Zenodo[27].

Synthetic data generation

The synthetic dataset was generated using the `generateSyntheticData()` function within the Katdetectr package. Mutations were randomly sampled on a reference genome such that each base has an equal probability, *p*, of being mutated (except for N bases for which *p* = 0). This reduces the occurrence of mutations on the reference genome to a sequence of $X_1, X_2, \ldots, X_n$, independent Bernoulli trials, X_i, i.e., a Bernoulli process, where;

$$\mathbf{P}(X_i = \mathbf{1}) = \mathbf{P}(\text{Mutation at } i\text{th base}) = p$$

$$\mathbf{P}(X_i = \mathbf{0}) = \mathbf{P}(\text{No mutation at } i\text{th base}) = 1 - p$$

Equation 7

with probability mass function (PMF), expectation and variance:

$$p_s(k) = \binom{n}{k} p^k (1 - p)^{n-k}, \quad k = 0, 1, \ldots., n$$

$$\mathbf{E}(S) = np$$

$$var(S) = np(1 - p)$$

Equation 8

with p as the probability of success (i.e., mutation), n as the number of independent trials (i.e., length of the genome in base pairs), and k as the number of successes (i.e., number of occurred mutations). The IMD now reduces to geometric random variable T; with PMF, expectation, and variance:

$$p_T(t) = (1 - p)^{-1}p$$

$$\mathbf{E}(T) = \frac{1}{p}$$

$$var(T) = \frac{1 - p}{p^2}$$

Equation 9

The genomic start location of a kataegis locus was sampled as an independent Bernoulli trial. The genomic end location of a kataegis locus was calculated using:

$$end_i = start_i + \mathbf{E}\,(T)_i(k_i + 1) - 1$$

Equation 10

Synthetic dataset description

The synthetic data consists of 1,024 samples with a total of 21,299,360 SNVs (**Table 2**). All mutations were generated on chromosome 1 on the human reference genome hg19. These samples were generated such that 8 different TMB classes (0.1, 0.5, 1, 5, 10, 50, 100, 500) were considered.

$$TMB = \frac{total\ number\ of\ variants\ in\ sample}{length\ of\ genome\ in\ bp} * 10^6$$

Equation 11

For each TMB class, a sample was generated for all combinations of the following parameters: the number of kataegis loci (1, 2, 3, 5); the number of variants within each kataegis loci (6, 10, 25, 50); and the expected IMD of the variants in kataegis loci (100, 250, 500, 750). This resulted in 64 kataegis samples per TMB class. To

balance the dataset, 64 samples without kataegis loci were generated for each TMB class. The synthetic dataset contained 1,232 kataegis loci and 33,245 variants within kataegis loci.

Descriptive statistics of synthetic dataset

TMB class (no. of background mutations)	No. Samples (with kataegis)	No. Kataegis loci	No. Variants in kataegis loci
0.1 (25)	128 (64)	176	4,005
0.5 (125)	128 (64)	176	4,,006
1 (249)	128 (64)	176	4006
5 (1,246)	128 (64)	176	4,014
10 (2,493)	128 (64)	176	4,,029
50 (12,463)	128 (64)	176	4077
100 (24,925)	128 (64)	176	4,183
500 (124,625)	128 (64)	176	4,925

Table 2. Showing per Tumor Mutational Burden (TMB) class: TMB, number of generated background mutations per sample, the total number of samples, total number of samples with kataegis, total number of kataegis loci, and total number of variants within a kataegis loci of 1024 synthetic samples.

Whole Genome Sequence (WGS) dataset description

The WGS dataset (as used in this study; **table 3**) is publicly available in .txt format[2]. This dataset contained 7,042 primary cancer samples from 30 different tissues; of which 507 originate from whole genome sequencing (WGS) and 6,535 from whole exome sequencing (WES). Only the WGS samples (n = 507) were originally labeled using a Piece-Wise Constant Fit (PCF) model and manually curated for kataegis presence (or absence) by the original study. Only the respective WGS samples, with a total of 3,382,751 SNVs, were re-interrogated within our performance evaluation. Additionally, we binned this dataset into three TMB classes (low: TMB < 0.1, middle: 0.1 ≥ TMB < 10, high: TMB ≥ 10) and filtered it such that it only contained single nucleotide variants (SNVs).

Descriptive statistics of WGS dataset.

TMB class	# Samples (with kataegis)	# Kataegis loci	# Variants in kataegis loci
Low: TMB < 0.1	301(45)	93	946
Middle: 0.1 ≥ TMB < 10	186 (89)	444	5,058
High: TMB ≥ 10	20(18)	336	3,107

Table 2. Showing per Tumor Mutational Burden (TMB) class: TMB range, the total number of samples, total number of samples with kataegis, total number of kataegis loci, and total number of variants within a kataegis loci of 507 Whole Genome Sequenced (WGS) samples labeled by Alexandrov *et al.* [1].

Pre-processing and parameter settings of alternative kataegis detection packages

Both the synthetic and the Alexandrov et al. datasets were converted to MAF format for use in MafTools [10] ClusteredMutations [11], and kataegis [12] and to BED format for use in SeqKat [13]. All other parameter settings for MafTools, kataegis, ClusteredMutations, and SeqKat were set to the default values as specified in their respective manuals and vignettes.

For SigProfilerClusters [14] both the synthetic and the Alexandrov et al. datasets were converted to a .txt file with column names as specified in the manual of SigProfilerClusters. We set the following parameters for SigProfilerSimulator(): genome="GRCh37", contexts = ['288'], simulations=100, overlap=True. For subsequent cluster detection, we set the following parameters for SigProfilerClusters.analysis(): genome="GRCh37", contexts="96", simContext=["288"], analysis="all", sortSims=True, subClassify=True, correction=True, calculateIMD=True, max_cpu=4, includedVAFs=False.

From the output of SigProfilerClusters we selected the class 2 (kataegis) clusters for further analysis. The definition of kataegis used by SigProfilerClusters differs from the one used in our performance evaluation. SigProfilerClusters defines kataegis as a cluster of ≥4 genomic variants of which the mean IMD is statistically different from the sample specific IMD cut-off. To include SigProfilerClusters in our performance evaluation we only selected clusters detected by SigProfilerClusters that fit the definition of kataegis we used for the performance evaluation, i.e., a kataegis locus contains ≥6 genomic variants with a mean IMD ≤1,000 bp.

Funding

This research received funding from the Daniel den Hoed Fonds - Cancer Computational Biology Center (DDHF-CCBC) grant.

Competing interests

None declared.

Data availability

All data used in the performance evaluation can be found on Zenodo[27]. All supporting data and materials are available in the *GigaScience* GigaDB database [28].

List of abbreviations

AMOC: At Most One Change, bp: base-pair, BinSeg: Binary Segmentation, IMD: Intermutation Distance, MAF: Mutation Annotation Format, MNV: Multi Nucleotide Variant, nMCC: normalized Matthews Correlation Coefficient, PCF: Piecewise Constant Fit, PELT: Pruned Exact Linear Time, SNV: Single Nucleotide Variant, SegNeigh: Segment Neighbourhoods, TMB: Tumor Mutational Burden, TNR: True Negative Rate, TPR: True Positive Rate, VCF: Variant Calling Format, WES: Whole Exome Sequencing, WGS: Whole Genome Sequencing.

Availability of supporting source code and requirements

- Project name: **Katdetectr**
- RRID: **SCR_023506**
- BiotoolsID: katdetectr
- Workflowhub: 10.48546/workflowhub.workflow.463.1
- Project home page:
    - https://bioconductor.org/packages/release/bioc/html/katdetectr.html

    - https://github.com/ErasmusMC-CCBC/katdetectr
- Operating system(s): Platform independent
- Programming language: R (>= 4.2)
- Other requirements:
  BiocParallel (>= 1.26.2), changepoint (>=2.2.3), checkmate (>= 2.0.0), dplyr (>= 1.0.8), GenomicRanges (>= 1.44.0), GenomeInfoDb (>= 1.28.4), IRanges (>=2.26.0), maftools (>= 2.10.5), methods (>= 4.1.3), rlang (>= 1.0.2), S4Vectors (>= 0.30.2), tibble (>= 3.1.6), VariantAnnotation (>= 1.38.0), Biobase (>= 2.54.0), Rdpack (>= 2.3.1), ggplot2 (>= 3.3.5), tidyr (>= 1.2.0), BSgenome (>= 1.62.0), ggtext (>= 0.1.1), BSgenome.Hsapiens.UCSC.hg19 (>= 1.4.3), BSgenome.Hsapiens.UCSC.hg38 (>= 1.4.4), plyranges (>= 1.17.0)
- License: GPL-3

- Project name: **Evaluation of Katdetectr and alternative kataegis detection packages**
- Workflowhub: 10.48546/workflowhub.workflow.500.1
- Project home page: https://github.com/ErasmusMC-CCBC/evaluation_katdetectr
- Operating system(s): Platform independent
- Programming language: R (>= 4.2)
- Other requirements: katdetectr (1.1.2), MafTools (2.13.0), ClusteredMutations (1.0.1), kataegis (0.99.2), SeqKat (0.0.8), SigProfilerClusters (1.0.11), dplyr (1.0.10), tidyr (1.2.1), ggplot2 (3.4.0), variantAnnotation (1.44.0), mltools (0.3.5)
- License: GPL-3

Author contributions

Daan M. Hazelaar: Conceptualization, Data curation, Formal Analysis, Investigation, Methodology, Software, Validation, Visualization, Writing – Original draft

Job van Riet: Conceptualization, Methodology, Investigation, Software, Visualization, Writing – review & editing

Youri Hoogstrate: Conceptualization, Methodology, Software, Writing - review & editing

Harmen J. G. van de Werken: Conceptualization, Funding acquisition, Investigation, Methodology, Project administration, Resources, Supervision, Writing - review & editing

Acknowledgments

We thank Martijn Lolkema, John Martens, Marcel Smid, Guido Jenster, and Stavros Makrodimitris for their discussions, input, and support. Additionally, we would like to thank Coen Berns and Yi Ping for their initial efforts in detecting kataegis.

References

[1] S. Nik-Zainal *et al.*, “Mutational Processes Molding the Genomes of 21 Breast Cancers,” *Cell*, vol. 149, no. 5, pp. 979–993, May 2012, doi: 10.1016/j.cell.2012.04.024.

[2] L. B. Alexandrov *et al.*, “Signatures of mutational processes in human cancer,” *Nature*, vol. 500, no. 7463, Art. no. 7463, Aug. 2013, doi: 10.1038/nature12477.

[3] P. J. Campbell *et al.*, “Pan-cancer analysis of whole genomes,” *Nature*, vol. 578, no. 7793, Art. no. 7793, Feb. 2020, doi: 10.1038/s41586-020-1969-6.

[4] L. B. Alexandrov *et al.*, “The repertoire of mutational signatures in human cancer,” *Nature*, vol. 578, no. 7793, Art. no. 7793, Feb. 2020, doi: 10.1038/s41586-020-1943-3.

[5] E. N. Bergstrom *et al.*, “Mapping clustered mutations in cancer reveals APOBEC3 mutagenesis of ecDNA,” *Nature*, vol. 602, no. 7897, Art. no. 7897, Feb. 2022, doi: 10.1038/s41586-022-04398-6.

[6] M. B. Burns *et al.*, “APOBEC3B is an enzymatic source of mutation in breast cancer,” *Nature*, vol. 494, no. 7437, Art. no. 7437, Feb. 2013, doi: 10.1038/nature11881.

[7] D. Mas-Ponte and F. Supek, “DNA mismatch repair promotes APOBEC3-mediated diffuse hypermutation in human cancers,” *Nat Genet*, vol. 52, no. 9, Art. no. 9, Sep. 2020, doi: 10.1038/s41588-020-0674-6.

[8] S.-Y. Lee, H. Wang, H. J. Cho, R. Xi, and T.-M. Kim, “The shaping of cancer genomes with the regional impact of mutation processes,” *Exp Mol Med*, vol. 54, no. 7, Art. no. 7, Jul. 2022, doi: 10.1038/s12276-022-00808-x.

[9] S. A. Roberts *et al.*, “Clustered Mutations in Yeast and in Human Cancers Can Arise from Damaged Long Single-Strand DNA Regions,” *Molecular Cell*, vol. 46, no. 4, pp. 424–435, May 2012, doi: 10.1016/j.molcel.2012.03.030.

[10] A. Mayakonda, D.-C. Lin, Y. Assenov, C. Plass, and H. P. Koeffler, “Maftools: efficient and comprehensive analysis of somatic variants in cancer,” *Genome Res.*, vol. 28, no. 11, pp. 1747–1756, Nov. 2018, doi: 10.1101/gr.239244.118.

[11] D. Lora, “ClusteredMutations: Location and Visualization of Clustered Somatic Mutations.” Apr. 29, 2016. Accessed: Nov. 28, 2022. [Online]. Available: https://CRAN.R-project.org/package=ClusteredMutations

[12] X. Lin *et al.*, “kataegis: an R package for identification and visualization of the genomic localized hypermutation regions using high-throughput sequencing,” *BMC Genomics*, vol. 22, no. 1, p. 440, Jun. 2021, doi: 10.1186/s12864-021-07696-x.

[13] F. Yousif, X. Lin, F. Fan, C. Lalansingh, and J. Macdonald, “SeqKat: Detection of Kataegis.” Mar. 11, 2020. Accessed: Nov. 28, 2022. [Online]. Available: https://CRAN.R-project.org/package=SeqKat

[14] E. N. Bergstrom, M. Kundu, N. Tbeileh, and L. B. Alexandrov, “Examining clustered somatic mutations with SigProfilerClusters,” *Bioinformatics*, vol. 38, no. 13, pp. 3470–3473, Jul. 2022, doi: 10.1093/bioinformatics/btac335.

[15] R. Killick, P. Fearnhead, and I. A. Eckley, “Optimal Detection of Changepoints With a Linear Computational Cost,” *Journal of the American Statistical Association*, vol. 107, no. 500, pp. 1590–1598, Dec. 2012, doi: 10.1080/01621459.2012.737745.

[16] A. J. Scott and M. Knott, “A Cluster Analysis Method for Grouping Means in the Analysis of Variance,” *Biometrics*, vol. 30, no. 3, pp. 507–512, 1974, doi: 10.2307/2529204.

[17] I. E. Auger and C. E. Lawrence, “Algorithms for the optimal identification of segment neighborhoods,” *Bulletin of Mathematical Biology*, vol. 51, no. 1, pp. 39–54, Jan. 1989, doi: 10.1016/S0092-8240(89)80047-3.

[18] P. Selenica *et al.*, “APOBEC mutagenesis, kataegis, chromothripsis in EGFR-mutant osimertinib-resistant lung adenocarcinomas,” *Annals of Oncology*, vol. 33, no. 12, pp. 1284–1295, Dec. 2022, doi: 10.1016/j.annonc.2022.09.151.

[19] A. Stenman, M. Yang, J. O. Paulsson, J. Zedenius, K. Paulsson, and C. C. Juhlin, “Pan-Genomic Sequencing Reveals Actionable CDKN2A/2B Deletions and Kataegis in Anaplastic Thyroid Carcinoma,” *Cancers*, vol. 13, no. 24, Art. no. 24, Jan. 2021, doi: 10.3390/cancers13246340.

[20] P. Priestley *et al.*, “Pan-cancer whole-genome analyses of metastatic solid tumours,” *Nature*, vol. 575, no. 7781, Art. no. 7781, Nov. 2019, doi: 10.1038/s41586-019-1689-y.

[21] D. M. Hazelaar and J. van Riet, “Characterization and Visualization of Kataegis in Sequencing Data. R package version 1.2.0.” [Online]. Available: https://doi.org/doi:10.18129/B9.bioc.katdetectr.

[22] J. Van Riet and D. Hazelaar, “ErasmusMC-CCBC/evaluation_katdetectr: Publication.” Zenodo, Sep. 08, 2023. doi: 10.5281/ZENODO.8328463.

[23] R Core Team, *R: A Language and Environment for Statistical Computing*. Vienna, Austria: R Foundation for Statistical Computing, 2022. [Online]. Available: https://www.R-project.org/

[24] R. Killick and I. Eckley, “changepoint:an R package for changepoint analysis,” *Journal of Statistical Software*, vol. 58, no. 3, Art. no. 3, 2014.

[25] R. Killick, K. Haynes, and I. A. Eckley, *changepoint: An R package for changepoint analysis software reference*. 2022. [Online]. Available: https://CRAN.R-project.org/package=changepoint

[26] D. Chicco and G. Jurman, “The advantages of the Matthews correlation coefficient (MCC) over F1 score and accuracy in binary classification evaluation,” *BMC Genomics*, vol. 21, no. 1, p. 6, Jan. 2020, doi: 10.1186/s12864-019-6413-7.

[27] D. Hazelaar, J. van Riet, and H. van de Werken, “Datasets used for the performance evaluation of kataegis detection tools.” Zenodo, Jun. 08, 2022. doi: 10.5281/ZENODO.8046959.

[28] Hazelaar DM; van Riet J; Hoogstrate Y; van de Werken HJG. Supporting data for "Katdetectr: An R/Bioconductor package utilizing unsupervised changepoint analysis for robust kataegis detection" GigaScience Database 2023. http://dx.doi.org/10.5524/102445

Figure 1

Click here to access/download;Figure;figure_1_overview_katdetectr.pdf

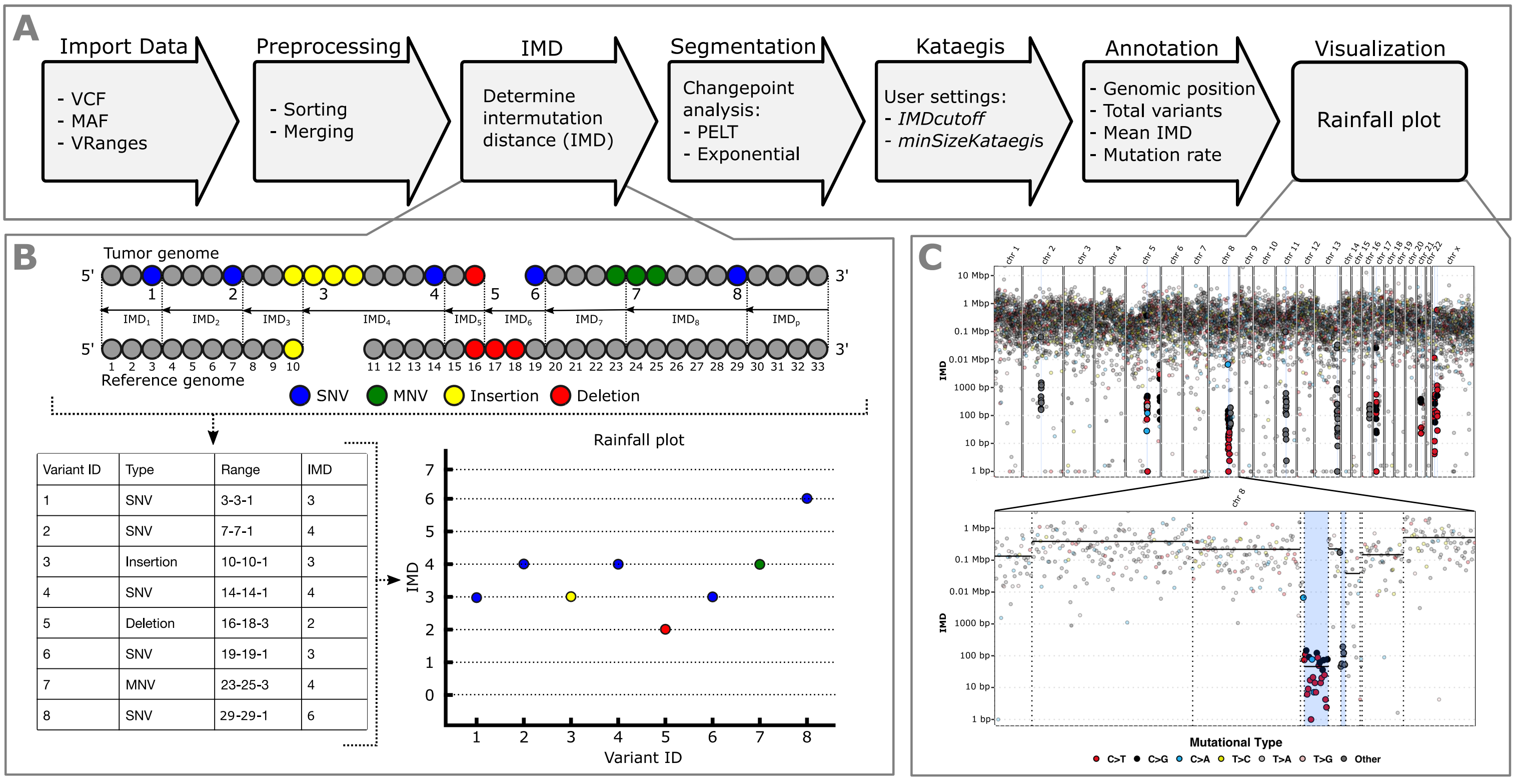

Variant ID	Type	Range	IMD
1	SNV	3-3-1	3
2	SNV	7-7-1	4
3	Insertion	10-10-1	3
4	SNV	14-14-1	4
5	Deletion	16-18-3	2
6	SNV	19-19-1	3
7	MNV	23-25-3	4
8	SNV	29-29-1	6

Click here to access/download;Figure;figure_3_rainfall_plots.pdf

A Synthetic sample 124625_1_50_100

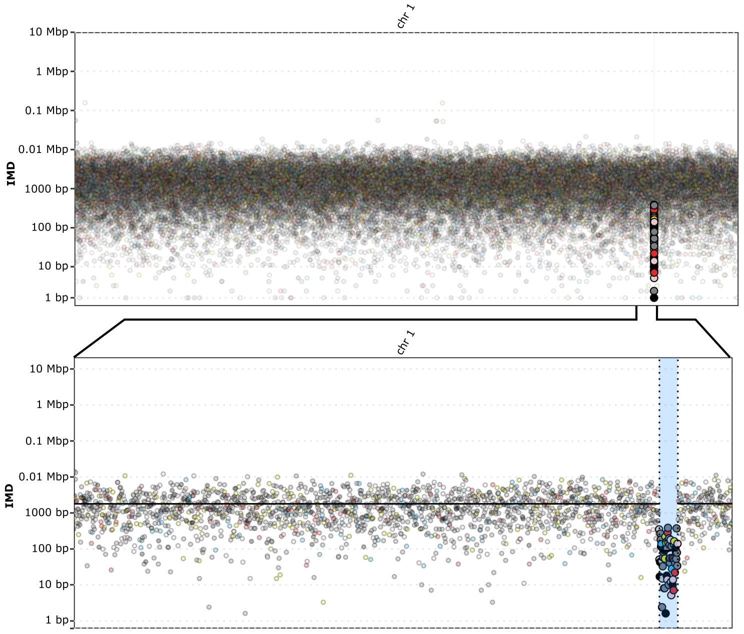

Mutational Type

C>T C>G C>A T>C T>A T>G Other

Confusion matrix

Package	TP	FP	TN	FN	Accuracy	nMCC
Katdetectr	57	0	124.618	0	1.00	1.00
SeqKat	57	25.959	98.659	0	0.79	0.52
MafTools	57	43.764	80.854	0	0.65	0.51
ClusteredMutations	57	49.581	75.037	0	0.60	0.51
SigProfilerClusters	57	57.814	66.804	0	0.54	0.51
Kataegis	0	0	124.618	57	0.99	0.50

B WGS sample LUAD-E01014

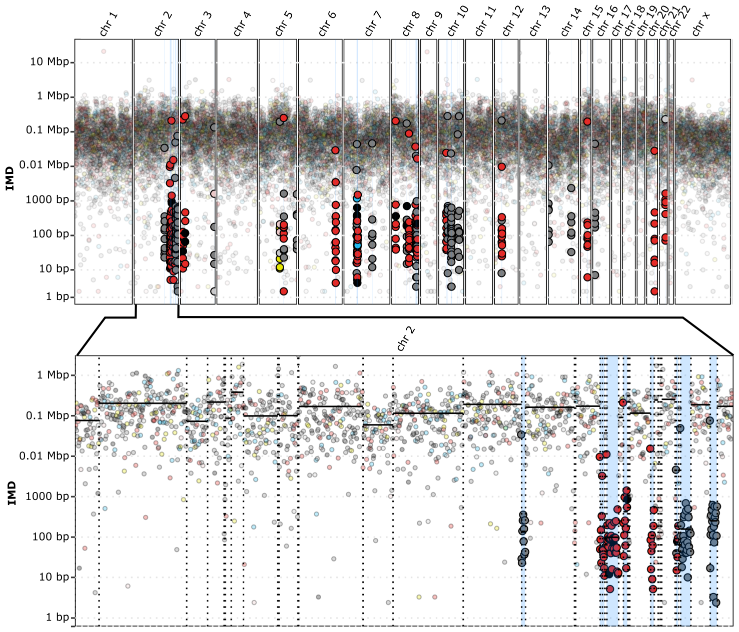

Mutational Type

C>T C>G C>A T>C T>A T>G Other

Confusion matrix

Package	TP	FP	TN	FN	Accuracy	nMCC
Katdetectr	399	50	22.925	1	0.99	0.97
MafTools	400	55	22.920	0	0.99	0.97
ClusteredMutations	400	72	22.903	0	0.99	0.96
SeqKat	253	37	22.938	147	0.99	0.87
Kataegis	0	0	22.975	400	0.98	0.50
SigProfilerClusters	0	0	22.975	400	0.98	0.50

C WGS sample PD7207a

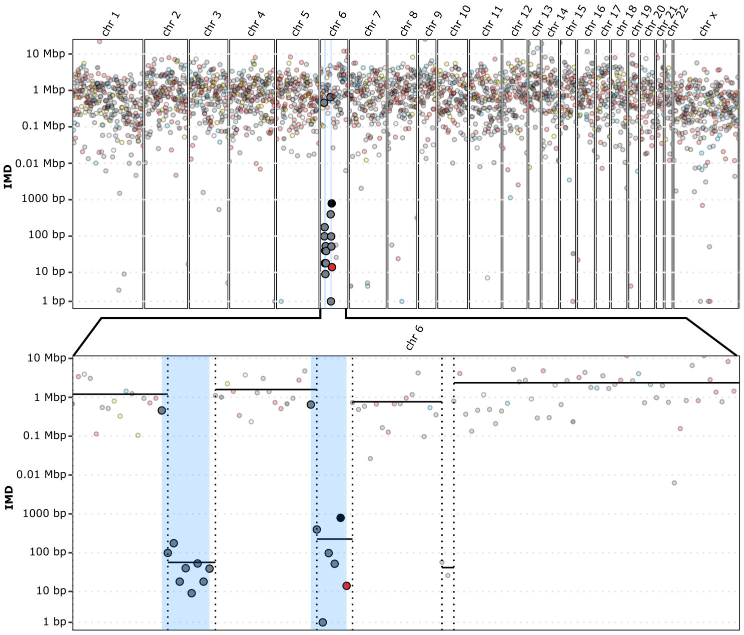

Mutational Type

C>T C>G C>A T>C T>A T>G Other

Confusion matrix

Package	TP	FP	TN	FN	Accuracy	nMCC
Katdetectr	14	2	2.568	0	0.99	0.97
Maftools	14	2	2.568	0	0.99	0.97
ClusteredMutations	14	2	2.568	0	0.99	0.97
SigProfilerClusters	14	2	2.568	0	0.99	0.97
SeqKat	0	0	2.570	14	0.99	0.50
Kataegis	0	0	2.570	14	0.99	0.50

D WGS sample PD4086a

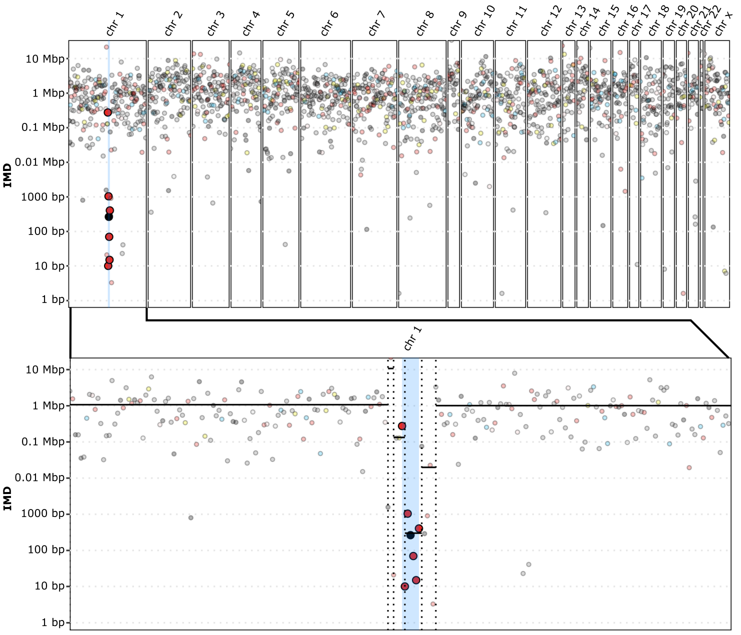

Mutational Type

C>T C>G C>A T>C T>A T>G Other

Confusion matrix

Package	TP	FP	TN	FN	Accuracy	nMCC
Katdetectr	0	7	1.922	0	0.99	0.50
SeqKat	0	7	1.922	0	0.99	0.50
MafTools	0	7	1.922	0	0.99	0.50
ClusteredMutations	0	7	1.922	0	0.99	0.50
SigProfilerClusters	0	7	1.922	0	0.99	0.50
Kataegis	0	0	1.929	0	1.00	0.50

Figure 2

Click here to access/download;Figure;figure_2_main_results_rebuttal.pdf

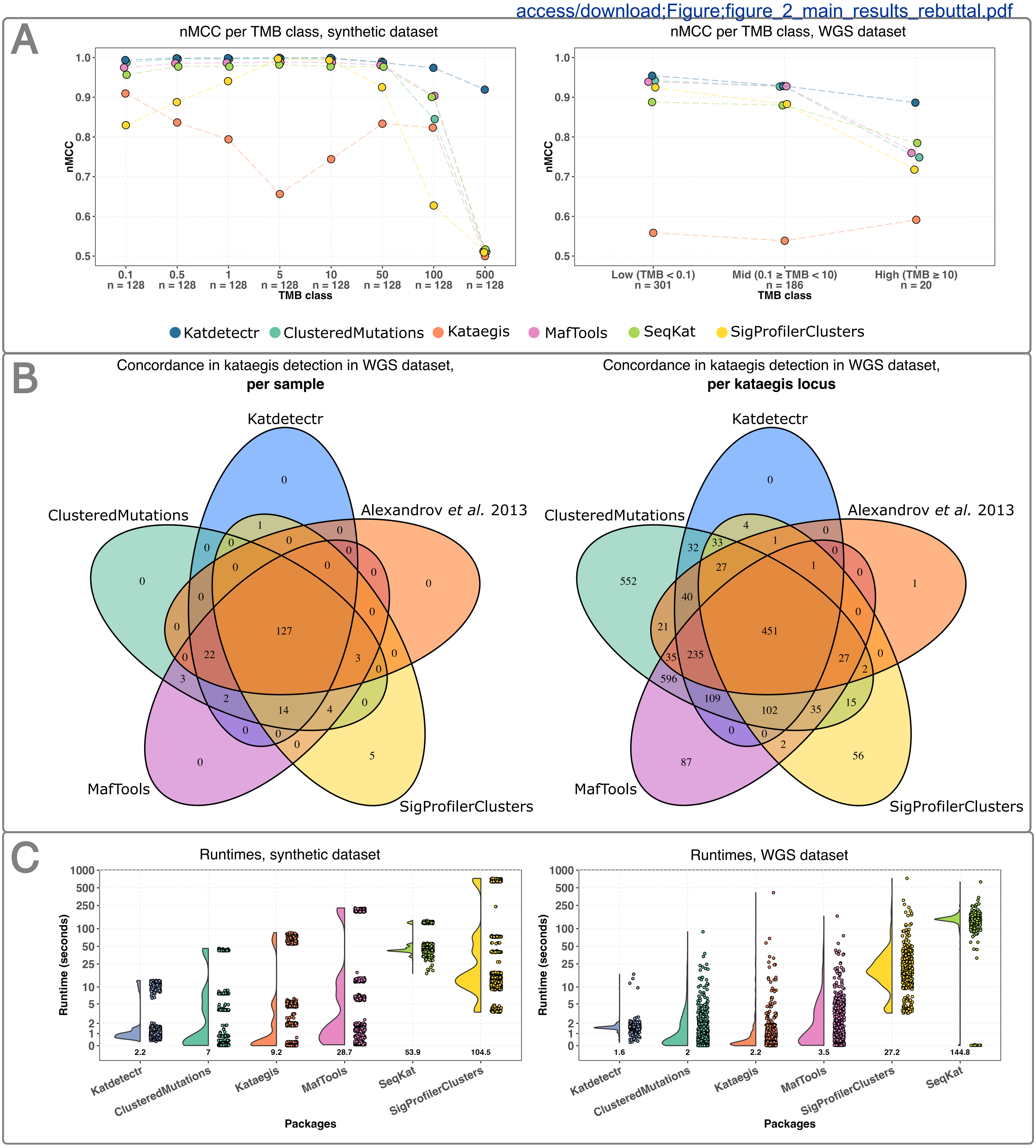

Click here to access/download

Supplementary Material

supplementary_material_vignette_general_overview.pdf

Supplementary Material Figure 1

Click here to access/download
Supplementary Material
supplementary_material_figure_1.docx

Supplementary Material Figure 2

Click here to access/download
Supplementary Material
supplementary_material_figure_2.docx

Supplementary Material Figure 3

Click here to access/download
Supplementary Material
supplemental_material_figure_3.docx

Supplementary Material Table 1

Click here to access/download
Supplementary Material
supplementary_material_table_1.docx

Supplementary Material Table 2

Click here to access/download
Supplementary Material
supplementary_material_table_2.docx

Rebuttal that contains mathemetical expressions

Click here to access/download
Supplementary Material
rebuttal_katdetectr_gigascience.pdf
