# Supplementary material for: Katdetectr: an R/bioconductor package utilizing unsupervised changepoint analysis for robust kataegis detection
Source: Gigascience. 2023 Oct 17;12:giad081. doi: 10.1093/gigascience/giad081 (PMC10580377; doi:10.1093/gigascience/giad081)
Supplement: giad081_Supplemental_Files [file giad081_supplemental_files.zip › supplementary_material_figure_1.docx]

**
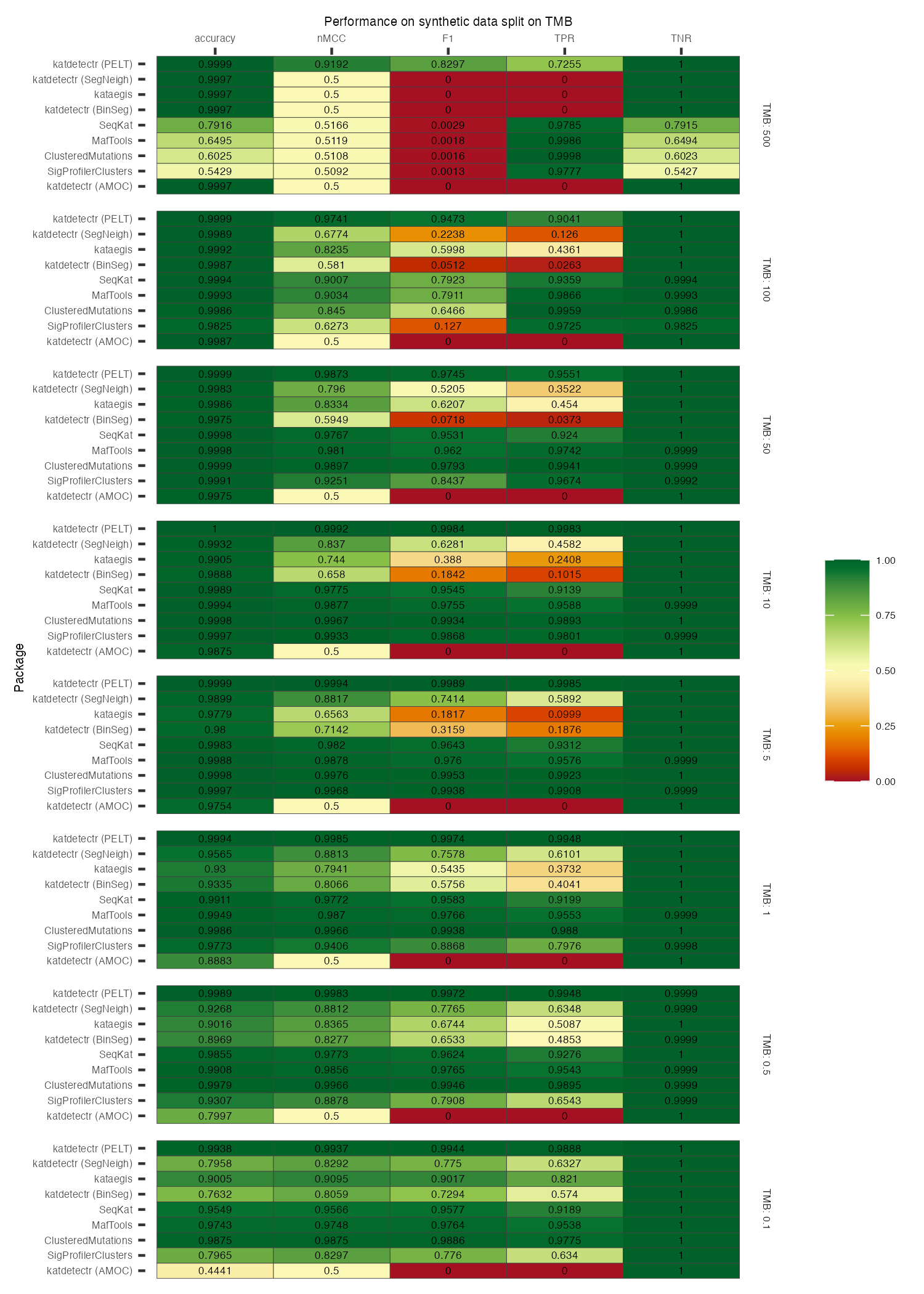
**

**Supplemental figure 1, Heatmap showing performance of kataegis detection packages on synthetic data.** Accuracy, normalized Matthews Correlation Coefficient (nMCC), F1 score, True Positive Rate (TPR) and True Negative Rate (TNR) for each of the Tumor Mutational Burden (TMB) Classes.
