# Supplementary material for: Katdetectr: an R/bioconductor package utilizing unsupervised changepoint analysis for robust kataegis detection
Source: Gigascience. 2023 Oct 17;12:giad081. doi: 10.1093/gigascience/giad081 (PMC10580377; doi:10.1093/gigascience/giad081)
Supplement: giad081_Supplemental_Files [file giad081_supplemental_files.zip › supplementary_material_figure_2.docx]

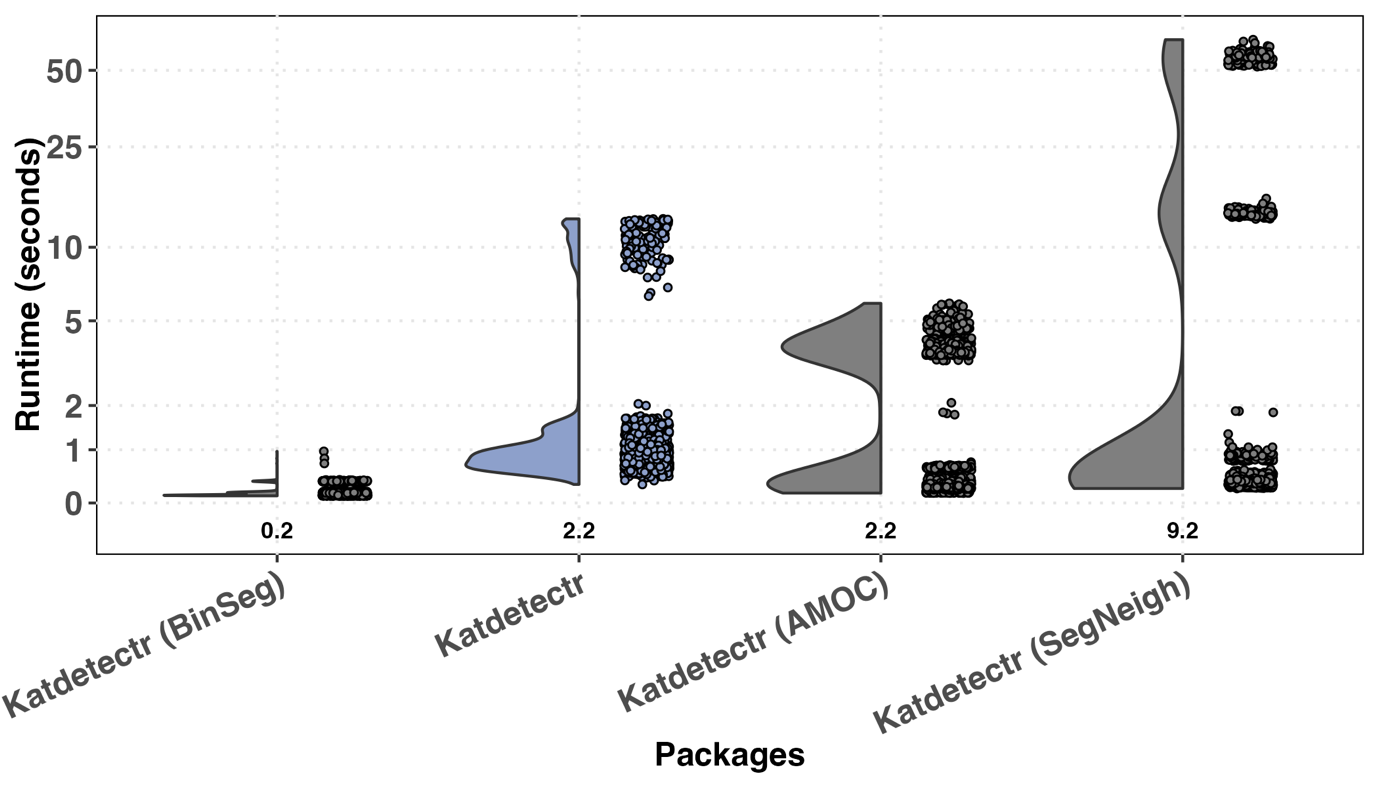


**Supplemental figure 2,** Violin plots with individual data-points representing the per sample runtimes of katdetectr using different search algorithms on the synthetic dataset. Boxplots were sorted in ascending order based on mean runtime (depicted in text below boxplot)
