# Supplementary material for: Katdetectr: an R/bioconductor package utilizing unsupervised changepoint analysis for robust kataegis detection
Source: Gigascience. 2023 Oct 17;12:giad081. doi: 10.1093/gigascience/giad081 (PMC10580377; doi:10.1093/gigascience/giad081)
Supplement: giad081_Supplemental_Files [file giad081_supplemental_files.zip › supplementary_material_table_1.docx]

**Supplementary table 1, confusion matrix for the synthetic dataset**

| **Package** | **True positive** | **False positive** | **True negative** | **False negative** |
| --- | --- | --- | --- | --- |
| Katdetectr (PELT) | 31.206 | 166 | 21.265.949 | 2.039 |
| Katdetectr (BinSeg) | 7.286 | 9 | 21.266.106 | 25.959 |
| Katdetectr (SegNeigh) | 13.656 | 10 | 21.166.415 | 19.279 |
| Katdetectr (AMOC) | 0 | 0 | 21.266.115 | 33.245 |
| Kataegis | 11.867 | 112 | 21.266.003 | 21.378 |
| SeqKat | 31.002 | 3.327.574 | 17.938.541 | 2.243 |
| MafTools | 32.194 | 5.594.792 | 15.671.323 | 1.051 |
| ClusteredMutations | 32.949 | 6.347.927 | 14.918.188 | 296 |
| SigProfilerClusters | 29.108 | 7.351.092 | 13.915.023 | 4.137 |
