# Supplementary material for: Katdetectr: an R/bioconductor package utilizing unsupervised changepoint analysis for robust kataegis detection
Source: Gigascience. 2023 Oct 17;12:giad081. doi: 10.1093/gigascience/giad081 (PMC10580377; doi:10.1093/gigascience/giad081)
Supplement: giad081_Supplemental_Files [file giad081_supplemental_files.zip › supplementary_material_table_2.docx]

**Supplementary table 2**, **confusion matrix for the WGS dataset.**

| **Package** | **True positive** | **False positive** | **True negative** | **False negative** |
| --- | --- | --- | --- | --- |
| Katdetectr | 8.256 | 2.667 | 3.370.973 | 855 |
| Kataegis | 160 | 16 | 3.373.624 | 8.951 |
| SeqKat | 5.396 | 1.092 | 3.372.548 | 3.715 |
| MafTools | 8.479 | 7.908 | 3.365.732 | 632 |
| ClusteredMutations | 9.049 | 11.341 | 3.362.299 | 62 |
| SigProfilerClusters | 6.026 | 2.595 | 3.371.045 | 3.085 |
