# Supplementary material for: Katdetectr: an R/bioconductor package utilizing unsupervised changepoint analysis for robust kataegis detection
Source: Gigascience. 2023 Oct 17;12:giad081. doi: 10.1093/gigascience/giad081 (PMC10580377; doi:10.1093/gigascience/giad081)
Supplement: giad081_Supplemental_Files [file giad081_supplemental_files.zip › supplementary_material_vignette_general_overview.pdf]

General overview Katdetectr

Daan Hazelaar

2023-02-24

Installation

To install this package, start R (version "4.2") and enter:

```
# Install via BioConductor
if (!require("BiocManager", quietly = TRUE))
    install.packages("BiocManager")

BiocManager::install("katdetectr")

# or the latest version
BiocManager::install("katdetectr", version = "devel")

# or from github
devtools::install_git("https://github.com/ErasmusMC-CCBC/katdetectr")
```

```
library(katdetectr)
```

Introduction

`katdetectr` is an *R* package for the detection, characterization and visualization of localized hypermutated regions, often referred to as *kataegis*.

The general workflow of `katdetectr` can be summarized as follows:

1. Import of genomic variants; VCF, MAF or VRanges objects.
2. Detection of kataegis foci.
3. Visualization of segmentation and kataegis foci.

Please see the Application Note (under submission) for additional background and details of `katdetectr`. The application note also contains a section regarding the performance of `katdetectr` and other kataegis detection packages: maftools, ClusteredMutations, SeqKat, kataegis, and SigProfilerClusters.

We have made `katdetectr` available on *BioConductor* as this insures reliability, and operability on common operating systems (Linux, Mac, and Windows). We designed `katdetectr` such that it fits well in the *BioConductor* ecosystem which allows `katdetectr` to be used easily in combination with other *BioConductor* packages and analysis pipelines.

Below, the `katdetectr` workflow is performed in a step-by-step manner on publicly-available datasets that are included within this package.

Importing genomic variants

Genomic variants from multiple common data-formats (VCF/MAF and VRanges objects) can be imported into `katdetectr`.

```
# Genomic variants stored within the VCF format.
pathToVCF <- system.file(package = "katdetectr", "extdata/CPTAC_Breast.vcf")

# Genomic variants stored within the MAF format.
pathToMAF <- system.file(package = "katdetectr", "extdata/APL_primary.maf")

# In addition, we can generate synthetic genomic variants including kataegis foci using generateSynthet
# This functions returns a VRanges object.
syntheticData <- generateSyntheticData(nBackgroundVariants = 2500, nKataegisFoci = 1)
```

Detection of kataegis foci

Using `detectKataegis()`, we can employ changepoint detection to detect distinct clusters of varying inter-mutation distance (IMD), mutation rate and size.

Imported genomic variant data can contain either single or multiple samples, in the latter case records can be aggregated by setting `aggregateRecords = TRUE`. Overlapping genomic variants (e.g., an InDel and SNV) are reduced into a single record.

From the genomic variants data the IMD is calculated. Following, changepoint analysis is performed on the IMD of the genomic variants which results in segments. Lastly, a segment is labelled as *kataegis foci* if the segment fits the following parameters: `minSizeKataegis = 6` and `IMDcutoff = 1000`.

```
# Detect kataegis foci within the given VCF file.
kdVCF <- detectKataegis(genomicVariants = pathToVCF)

# # Detect kataegis foci within the given MAF file.
# As this file contains multiple samples, we set aggregateRecords = TRUE.
kdMAF <- detectKataegis(genomicVariants = pathToMAF, aggregateRecords = TRUE)

# Detect kataegis foci within the synthetic data.
kdSynthetic <- detectKataegis(genomicVariants = syntheticData)
```

All relevant input and subsequent results are stored within `KatDetect` objects. Using `summary()`, `show()` and/or `print()`, we can generate overviews of these `KatDetect` object(s).

```
summary(kdVCF)
```

```
## Sample name:                              CPTAC
## Total number of genomic variants:         3684
## Total number of putative Kataegis foci:   9
## Total number of variants in a Kataegis foci: 133
```

```
print(kdVCF)
```

```
## Sample name:                                 CPTAC
## Total number of genomic variants:            3684
## Total number of putative Kataegis foci:      9
## Total number of variants in a Kataegis foci: 133
```

```r
show(kdVCF)
```

```
## Class 'KatDetect' : KatDetect Object
##                   : S4 class containing 4 slots with names:
##                     kataegisFoci genomicVariants segments info
##
## Created on:         Fri Feb 24 12:58:35 2023
## katdetectr version: 1.1.3
##
## summary:
## --------------------------------------------------------
## Sample name:                                 CPTAC
## Total number of genomic variants:            3684
## Total number of putative Kataegis foci:      9
## Total number of variants in a Kataegis foci: 133
## --------------------------------------------------------
```

```r
# Or simply:
kdVCF
```

```
## Class 'KatDetect' : KatDetect Object
##                   : S4 class containing 4 slots with names:
##                     kataegisFoci genomicVariants segments info
##
## Created on:         Fri Feb 24 12:58:35 2023
## katdetectr version: 1.1.3
##
## summary:
## --------------------------------------------------------
## Sample name:                                 CPTAC
## Total number of genomic variants:            3684
## Total number of putative Kataegis foci:      9
## Total number of variants in a Kataegis foci: 133
## --------------------------------------------------------
```

Underlying data can be retrieved from a `KatDetect` objects using the following getter functions:

1. `getGenomicVariants()` returns: VRanges object. Processed genomic variants used as input for changepoint detection. This VRanges contains the genomic location, IMD, and kataegis status of each genomic variant
2. `getSegments()` returns: GRanges object. Contains the segments as derived from changepoint detection. This Granges contains the genomic location, total number of variants, mean IMD and, mutation rate of each segment.
3. `getKataegisFoci()` returns: GRanges object. Contains all segments designated as putative kataegis foci according the the specified parameters (minSizeKataegis and IMDcutoff). This Granges contains the genomic location, total number of variants and mean IMD of each putative kataegis foci
4. `getInfo()` returns: List object. Contains supplementary information including used parameter settings.

```
getGenomicVariants(kdVCF)
```

```
## VRanges object with 3684 ranges and 5 metadata columns:
##          seqnames    ranges strand         ref              alt     totalDepth
##             <Rle> <IRanges>  <Rle> <character> <characterOrRle> <integerOrRle>
##      [1]     chr1    935222      *           C                A             50
##      [2]     chr1    949608      *           G                A             50
##      [3]     chr1    981131      *           A                G             50
##      [4]     chr1    982722      *           A                G             50
##      [5]     chr1   1164015      *           C                A             50
##      ...      ...       ...    ...         ...              ...            ...
##   [3680]     chrX 153594977      *           G                A             50
##   [3681]     chrX 153627839      *           C                T             50
##   [3682]     chrX 153629155      *           A                G             50
##   [3683]     chrX 153668757      *           G                A             50
##   [3684]     chrX 153764217      *           C                T             50
##                refDepth       altDepth   sampleNames softFilterMatrix | revmap
##          <integerOrRle> <integerOrRle> <factorOrRle>         <matrix> | <list>
##      [1]             20             30         CPTAC                  |      1
##      [2]             20             30         CPTAC                  |      2
##      [3]             20             30         CPTAC                  |      3
##      [4]             20             30         CPTAC                  |      4
##      [5]             20             30         CPTAC                  |      5
##      ...            ...            ...           ...              ... .    ...
##   [3680]             20             30         CPTAC                  |   3683
##   [3681]             20             30         CPTAC                  |   3684
##   [3682]             20             30         CPTAC                  |   3685
##   [3683]             20             30         CPTAC                  |   3686
##   [3684]             20             30         CPTAC                  |   3687
##          variantID       IMD segmentID putativeKataegis
##          <integer> <integer> <integer>        <logical>
##      [1]         1    935222         1            FALSE
##      [2]         2     14386         1            FALSE
##      [3]         3     31523         1            FALSE
##      [4]         4      1591         1            FALSE
##      [5]         5    181293         1            FALSE
##      ...       ...       ...       ...              ...
##   [3680]      3680       442         5            FALSE
##   [3681]      3681     32862         6            FALSE
##   [3682]      3682      1316         6            FALSE
##   [3683]      3683     39602         6            FALSE
##   [3684]      3684     95460         7            FALSE
##   -------
##   seqinfo: 23 sequences from an unspecified genome; no seqlengths
##   hardFilters: NULL
```

```
getSegments(kdVCF)
```

```
## GRanges object with 452 ranges and 8 metadata columns:
##         seqnames             ranges strand | segmentID totalVariants
##            <Rle>          <IRanges>  <Rle> | <numeric>     <numeric>
##     [1]     chr1          1-3389727      * |         1            11
```

```
##     [2]     chr1     3389728-3428608      * |         2            4
##     [3]     chr1    3428609-19199400      * |         3           22
##     [4]     chr1   19199401-19203725      * |         4            4
##     [5]     chr1   19203726-19635011      * |         5            8
##     ...      ...                 ...    ... .       ...          ...
##   [448]     chrX 153577919-153594977      * |         5            6
##   [449]     chrX 153594978-153668757      * |         6            3
##   [450]     chrX 153668758-155270560      * |         7            1
##   [451]     chrY          1-59373566      * |         1            0
##   [452]     chrM             1-16571      * |         1            0
##         firstVariantID lastVariantID    meanIMD mutationRate sampleNames
##              <integer>     <integer>  <numeric>    <numeric> <character>
##     [1]              1            11  308157.00  3.24510e-06       CPTAC
##     [2]             12            15    9720.25  1.02878e-04       CPTAC
##     [3]             16            37  716854.18  1.39498e-06       CPTAC
##     [4]             38            41    1081.25  9.24855e-04       CPTAC
##     [5]             42            49   53910.75  1.85492e-05       CPTAC
##     ...            ...           ...        ...          ...         ...
##   [448]           3675          3680    2843.17  3.51720e-04       CPTAC
##   [449]           3681          3683   24593.33  4.06614e-05       CPTAC
##   [450]           3684          3684 1601802.00  6.24297e-07       CPTAC
##   [451]           <NA>          <NA>         NA  0.00000e+00       CPTAC
##   [452]           <NA>          <NA>         NA  0.00000e+00       CPTAC
##         IMDcutoffValues
##               <numeric>
##     [1]            1000
##     [2]            1000
##     [3]            1000
##     [4]            1000
##     [5]            1000
##     ...             ...
##   [448]            1000
##   [449]            1000
##   [450]            1000
##   [451]            1000
##   [452]            1000
##   -------
##   seqinfo: 25 sequences from an unspecified genome; no seqlengths
```

```
getKataegisFoci(kdVCF)
```

```
## GRanges object with 9 ranges and 7 metadata columns:
##       seqnames              ranges strand |    fociID sampleNames totalVariants
##          <Rle>           <IRanges>  <Rle> | <integer> <character>     <numeric>
##   [1]     chr3   58108856-58111467      * |         1       CPTAC             7
##   [2]     chr6   32489708-32489949      * |         2       CPTAC            13
##   [3]     chr6   32632598-32632770      * |         3       CPTAC             8
##   [4]     chr6 151669875-151674326      * |         4       CPTAC             7
##   [5]     chr8 144991205-144999107      * |         5       CPTAC            25
##   [6]    chr11   62285208-62298597      * |         6       CPTAC            25
##   [7]    chr14 105405599-105419557      * |         7       CPTAC            23
##   [8]    chr15   86122654-86124712      * |         8       CPTAC             6
##   [9]    chr19     4510560-4513559      * |         9       CPTAC            19
##       firstVariantID lastVariantID   meanIMD IMDcutoff
```

```
##             <numeric>     <integer> <numeric> <numeric>
##   [1]             782           788  435.1667      1000
##   [2]            1251          1263   20.0833      1000
##   [3]            1273          1280   24.5714      1000
##   [4]            1358          1364  741.8333      1000
##   [5]            1659          1683  329.2500      1000
##   [6]            2112          2136  557.8750      1000
##   [7]            2591          2613  634.4545      1000
##   [8]            2687          2692  411.6000      1000
##   [9]            3139          3157  166.6111      1000
##   -------
##   seqinfo: 25 sequences from an unspecified genome; no seqlengths
```

```
getInfo(kdVCF)
```

```
## $sampleName
## [1] "CPTAC"
##
## $totalGenomicVariants
## [1] 3684
##
## $totalKataegisFoci
## [1] 9
##
## $totalVariantsInKataegisFoci
## [1] 133
##
## $version
## [1] "1.1.3"
##
## $date
## [1] "Fri Feb 24 12:58:35 2023"
##
## $parameters
## $parameters$minSizeKataegis
## [1] 6
##
## $parameters$IMDcutoff
## [1] 1000
##
## $parameters$test.stat
## [1] "Exponential"
##
## $parameters$penalty
## [1] "BIC"
##
## $parameters$pen.value
## [1] 0
##
## $parameters$method
## [1] "PELT"
##
## $parameters$minseglen
## [1] 2
```

```
##
## $parameters$aggregateRecords
## [1] FALSE
```

Visualization of segmentation and kataegis foci

Per sample, we can visualize the IMD, detected segments and putative kataegis foci as a rainfall plot. In addition, this allows for a per-chromosome approach which can highlight the putative kataegis foci.

```
rainfallPlot(kdVCF)
```

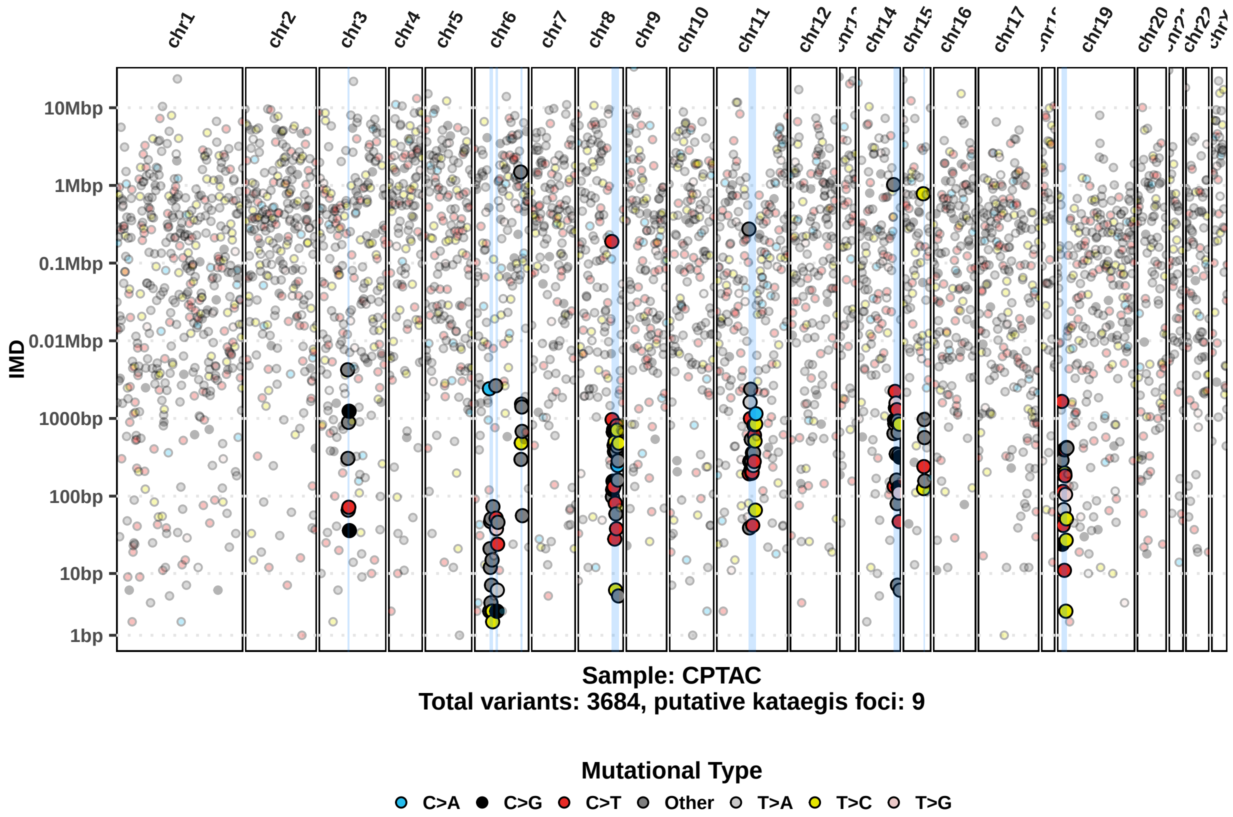


```
# With showSegmentation, the detected segments (changepoints) as visualized with their mean IMD.
rainfallPlot(kdMAF, showSegmentation = TRUE)
```

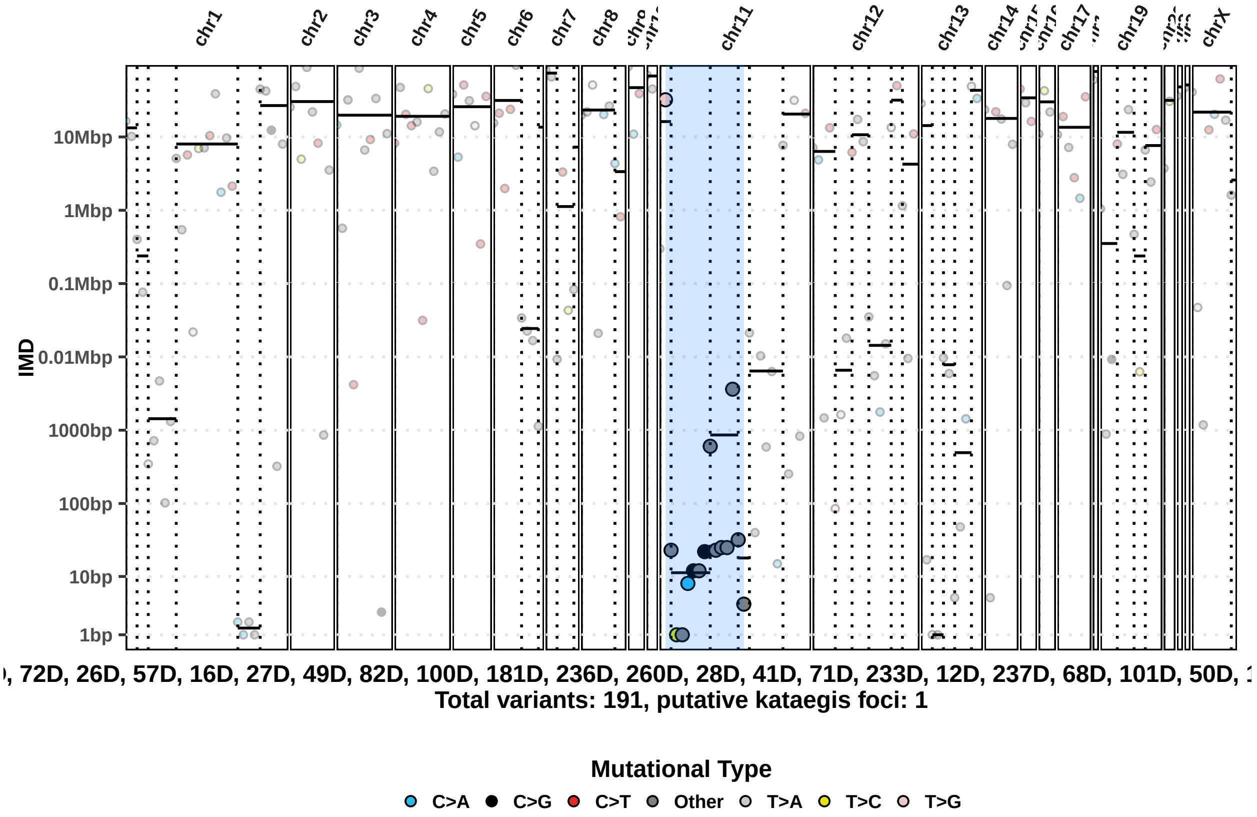


```
# With showSequence, we can display specific chromosomes or all chromosomes in which a putative kataegi
rainfallPlot(kdSynthetic, showKataegis = TRUE, showSegmentation = TRUE, showSequence = "Kataegis")
```

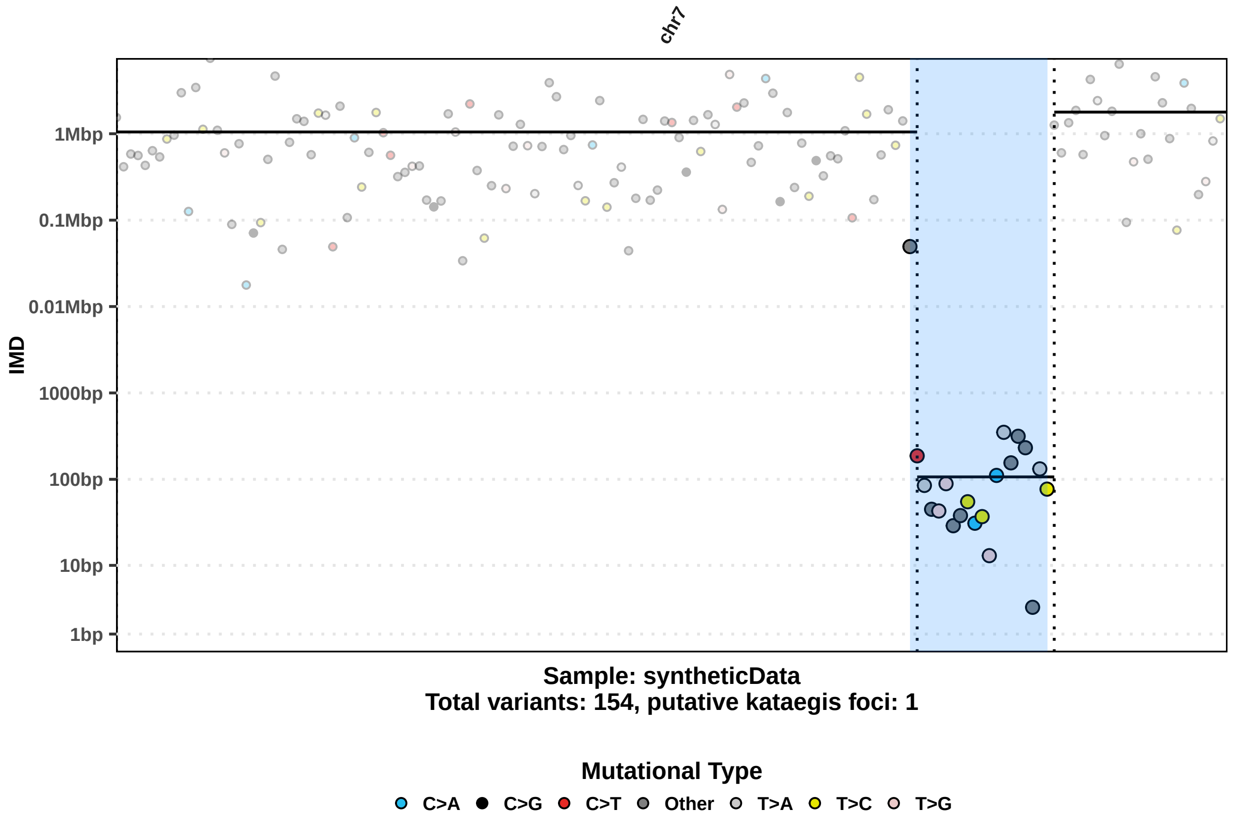

More parameter settings

`katdetectr` has been implemented flexibly which allows its users to detect clustered mutations of different classes. The historical definition of kataegis foci, postulated by Alexandrov et al.* is a segment harboring more than 6 variants and has a mean IMD less than 1000bp. However, these parameters can be set differently in `detectKataegis()`.

For example, another type of clustered mutations are called Omikli: a segment harboring 2 or 3 variants with mean IMD = m

Note that we did not evaluate the performance of `katdetectr` in regards to detecting other types of clusters. The following is just to show you how to change the parameters of `detectKataegis()` if you want to use `katdetectr` for detecting these types of clusters.

```
# detect putative Omikli, size 3 and mean IMD = 500
kdSyntheticOmikli500 <- detectKataegis(genomicVariants = syntheticData, minSizeKataegis = 3, IMDcutoff =

# detect putative Omikli, size 4 and mean IMD = 1000
kdSyntheticOmikli500 <- detectKataegis(genomicVariants = syntheticData, minSizeKataegis = 4, IMDcutoff =
```

We tested katdetectr with multiple parameter settings (test.stat, penalty, pen.value, minseglen) in order to obtain the highest performance in regards to kataegis classification. The best combination of parameters have been set as the default values. We recommend using these parameter settings! For more information regarding the these parameters for changepoint analysis see Killick2014

Custom function for IMD cutoff value.

As show previously, the IMD cutoff value can be set by the user in order to identify different types of mutation clusters. However, it is also possible to define a custom function that determines a IMD cutoff value for each detected segment. This flexibility allow you to define your own kataegis definition while still using the `katdetectr` framework. Below we show how to implement a custom function for the IMD cutoff value. The function below comes from the work of Pan-Cancer Analysis of Whole Genomes Consortium

```
# function for modeling sample mutation rate
modelSampleRate <- function(IMDs){

    lambda <- log(2) / median(IMDs)

    return(lambda)
}

# function for calculating the nth root of x
nthroot = function(x, n){

    y <- x^(1 / n)

    return(y)
}

# Function that defines the IMD cutoff specific for each segment
# Within this function you can use all variables available in the slots: genomicVariants and segments
IMDcutoffFun <- function(genomicVariants, segments){

    IMDs <- genomicVariants$IMD
    totalVariants <-  segments$totalVariants
    width <- segments |> dplyr::as_tibble() |> dplyr::pull(width)

    sampleRate <- modelSampleRate(IMDs)

    IMDcutoff <- -log(1 - nthroot(0.01 / width, ifelse(totalVariants != 0, totalVariants - 1, 1))) / sa

    IMDcutoff <- replace(IMDcutoff, IMDcutoff > 1000, 1000)

    return(IMDcutoff)
}

kdCustom <- detectKataegis(syntheticData, IMDcutoff = IMDcutoffFun)
```

Analyzing non standard sequences

The human autosomes and sex chromosomes from the reference genome hg19 and hg38 come implemented in `katdetectr`. However, other (non standard) sequences can be analyzed if the correct arguments are provided. You have to provide a dataframe that contains the length of all the sequences you want to analyze.

```
# generate data that contains non standard sequences
syndata1 <- generateSyntheticData(seqnames = c("chr1_gl000191_random", "chr4_ctg9_hap1"))
syndata2 <- generateSyntheticData(seqnames = "chr1")
```

```r
syndata <- suppressWarnings(c(syndata1, syndata2))

# construct a dataframe that contains the length of the sequences
# each column name (name of the sequence)
sequenceLength = data.frame(
    chr1 = 249250621,
    chr1_gl000191_random = 106433,
    chr4_ctg9_hap1 = 590426
)

# provide the dataframe with the sequence lengths using the refSeq argument
kdNonStandard <- detectKataegis(genomicVariants = syndata, refSeq = sequenceLength)
```

Session Information

```r
utils::sessionInfo()
```

```
## R version 4.2.0 (2022-04-22)
## Platform: aarch64-apple-darwin20 (64-bit)
## Running under: macOS 13.2
##
## Matrix products: default
## BLAS:   /Library/Frameworks/R.framework/Versions/4.2-arm64/Resources/lib/libRblas.0.dylib
## LAPACK: /Library/Frameworks/R.framework/Versions/4.2-arm64/Resources/lib/libRlapack.dylib
##
## locale:
## [1] en_US.UTF-8/en_US.UTF-8/en_US.UTF-8/C/en_US.UTF-8/en_US.UTF-8
##
## attached base packages:
## [1] stats     graphics  grDevices utils     datasets  methods   base
##
## other attached packages:
## [1] katdetectr_1.1.3
##
## loaded via a namespace (and not attached):
##   [1] ggtext_0.1.2                     bitops_1.0-7
##   [3] matrixStats_0.63.0               bit64_4.0.5
##   [5] filelock_1.0.2                   RColorBrewer_1.1-3
##   [7] progress_1.2.2                   httr_1.4.4
##   [9] GenomeInfoDb_1.34.9              tools_4.2.0
##  [11] backports_1.4.1                  utf8_1.2.3
##  [13] R6_2.5.1                         colorspace_2.1-0
##  [15] DBI_1.1.3                        BiocGenerics_0.44.0
##  [17] DNAcopy_1.72.3                   withr_2.5.0
##  [19] tidyselect_1.2.0                 prettyunits_1.1.1
##  [21] bit_4.0.5                        curl_5.0.0
##  [23] compiler_4.2.0                   cli_3.6.0
##  [25] Biobase_2.58.0                   xml2_1.3.3
##  [27] DelayedArray_0.24.0              labeling_0.4.2
##  [29] rtracklayer_1.58.0               scales_1.2.1
```

```
##  [31] checkmate_2.1.0                   rappdirs_0.3.3
##  [33] commonmark_1.8.1                  stringr_1.5.0
##  [35] digest_0.6.31                     Rsamtools_2.14.0
##  [37] rmarkdown_2.20                    BSgenome.Hsapiens.UCSC.hg19_1.4.3
##  [39] XVector_0.38.0                    pkgconfig_2.0.3
##  [41] htmltools_0.5.4                   changepoint_2.2.4
##  [43] MatrixGenerics_1.10.0             highr_0.10
##  [45] dbplyr_2.3.0                      fastmap_1.1.0
##  [47] BSgenome_1.66.3                   rlang_1.0.6
##  [49] rstudioapi_0.14                   RSQLite_2.3.0
##  [51] farver_2.1.1                      BiocIO_1.8.0
##  [53] generics_0.1.3                    zoo_1.8-11
##  [55] BiocParallel_1.32.5               dplyr_1.1.0
##  [57] VariantAnnotation_1.44.1          RCurl_1.98-1.10
##  [59] magrittr_2.0.3                    GenomeInfoDbData_1.2.9
##  [61] Matrix_1.5-3                      munsell_0.5.0
##  [63] Rcpp_1.0.10                       S4Vectors_0.36.1
##  [65] fansi_1.0.4                       lifecycle_1.0.3
##  [67] stringi_1.7.12                    yaml_2.3.7
##  [69] SummarizedExperiment_1.28.0       zlibbioc_1.44.0
##  [71] BiocFileCache_2.6.1               grid_4.2.0
##  [73] blob_1.2.3                        parallel_4.2.0
##  [75] crayon_1.5.2                      lattice_0.20-45
##  [77] splines_4.2.0                     Biostrings_2.66.0
##  [79] gridtext_0.1.5                    GenomicFeatures_1.50.4
##  [81] hms_1.1.2                         KEGGREST_1.38.0
##  [83] BSgenome.Hsapiens.UCSC.hg38_1.4.5 knitr_1.42
##  [85] pillar_1.8.1                      GenomicRanges_1.50.2
##  [87] rjson_0.2.21                      markdown_1.5
##  [89] codetools_0.2-19                  biomaRt_2.54.0
##  [91] stats4_4.2.0                      XML_3.99-0.13
##  [93] glue_1.6.2                        evaluate_0.20
##  [95] data.table_1.14.8                 png_0.1-8
##  [97] vctrs_0.5.2                       Rdpack_2.4
##  [99] gtable_0.3.1                      purrr_1.0.1
## [101] tidyr_1.3.0                       assertthat_0.2.1
## [103] ggplot2_3.4.1                     cachem_1.0.6
## [105] xfun_0.37                         rbibutils_2.2.13
## [107] restfulr_0.0.15                   survival_3.5-3
## [109] tibble_3.1.8                      GenomicAlignments_1.34.0
## [111] AnnotationDbi_1.60.0              plyranges_1.18.0
## [113] memoise_2.0.1                     IRanges_2.32.0
## [115] maftools_2.14.0                   ellipsis_0.3.2
```
