# Supplementary material for: Katdetectr: an R/bioconductor package utilizing unsupervised changepoint analysis for robust kataegis detection
Source: Gigascience. 2023 Oct 17;12:giad081. doi: 10.1093/gigascience/giad081 (PMC10580377; doi:10.1093/gigascience/giad081)
Supplement: giad081_Response_to_Reviewer_Comments_Original_Submission [file giad081_response_to_reviewer_comments_original_submission.pdf]

Author's Response To Reviewer Comments

Please note that we have added a supplementary pdf file that contains our response to the editor and the reviewers. This supplementary files contains mathematical expressions which we use in our response to the reviewers.

Concerning: GIGA-D-23-00051 and detailed response to its review

Dear Hongling Zhou,

Thank you very much for the thorough evaluation of our manuscript GIGA-D-23-00051 entitled: Katdetectr: An R/Bioconductor package utilizing unsupervised changepoint analysis for robust kataegis detection" by Daan Hazelaar; Job van Riet; Youri Hoogstrate; Harmen van de Werken.

We greatly appreciate the opportunity to revise our manuscript according to the high-quality reports of the reviewers. We include a point-by-point reply to the criticism and suggestions by the reviewers and you. Moreover, the described changes are indicated with track changes in the resubmitted manuscript.

1. Register any new software application in the bio.tools and SciCrunch.org databases to receive RRID (Research Resource Identification Initiative ID) and biotoolsID identifiers, and include these in your manuscript.

Dear dr. Hongling Zhou, we have registered katdetectr on bio.tools (biotoolsID: katdetectr)) and SciCrunch.org (RRID: SCR_023506) and added the accompanying identifiers to the manuscript under the section: Availability and requirements in compliance with the GIGA journal requirements.

2. Computational workflows should be registered in workflowhub.eu and the DOIs cited in the relevant places in the manuscript.

We have registered katdetectr (10.48546/workflowhub.workflow.463.1) and the performance evaluation of katdetectr (10.48546/workflowhub.workflow.500.1) on workflowhub.eu and added the corresponding to the availability and requirements section in the manuscript.

Sincerely yours,

Harmen J. G. van de Werken, Ph.D.
Assistant Professor in Computational Biology & Bioinformatics in Immunology and Cancer at the Erasmus Medical Center in the Department of Immunology

Reviewer #1: minor revision
In this short manuscript, Hazelaar et al. describe a new software package written in R, called "katdetectr". This package can be useful as an addition to existing computational tools for identifying and characterizing kataegis in cancer genomes. The paper then compares katdetectr favorably against other software for

detecting kataegis, using synthetic and real cancer data. Overall, the paper is fine and the katdetectr package is a nice addition for researchers' toolbox. I would suggest that the authors make the following improve-ments.

1. Choose a convention for decimal point and digit separator, then stick with it. "." was used as both the decimal point and digit separator for large numbers, which gets confusing. Typically, "." Is used for decimal point and "," is used for digit separator.

We thank the reviewer for this editorial comment. We have indeed revised our manuscript (and figures) in accordance the convention of using "." as a decimal point and using "," as a digit separator. We apologize for the previous oversight.

2. The Introduction is so abbreviated that it doesn't serve much purpose. Either flesh it out with more information or just drop it completely. This journal accepts papers that go right into re-sults, so it's fine. But the authors should also consider if writing a more expansive introduction can make the paper more accessible to readers who aren't already as knowledgeable.

According to the reviewer's suggestion, we have extended the introduction to improve this manu-script's accessibility for readers not yet familiar with kataegis. Additionally, we have included additional references within the introduction to promote further reading into the current state of re-search regarding kataegis (lines 60 - 78).

3. The biggest issue is using the 2013 Alexandrov kataegis calls as "ground truth" when multi-ple packages published since then detect 102 loci that Alexandrov (2013) missed. Seems like it would be much more sensible to use the calls from the 2020 PCAWG paper instead: https://doi.org/10.1038/s41586-020-1969-6. The data are controlled access, but it should be possible to get them.

Whilst we agree with the reviewer that utilizing the latest release of the kataegis calls (as called within the PCAWG) would be a worthwhile endeavor as the PCAWG-calls would indeed be more recent and potentially contain improved annotations. However, this dataset is currently (as mentioned) only available under controlled-access whilst the Alexandrov et al. call-set is publicly available.

In line with the philosophy of open science and Giga Science, we believe that reproducible and continued benchmarking of novel computational methodology against comparable methodology is paramount and that this is restricted when controlled-access data in involved.

Therefore, we used the publicly-available dataset as described by Alexandrov et al. (2013) for benchmarking which allowed us to co-publish our input data and results for public review and future comparison without restriction.

To overcome the potential inaccuracy of the employed ground-truth call-set, we compared the evaluated methodologies without the dependence of the "ground-truth" labels by employing a Venn diagram (Fig. 2b) which highlights the (shared) dis/concordance against the "ground-truth". This allows for a visual comparison of the packages which is less dependent on the input.

We have extended and refined our discussion to address these valid concerns on the employed "ground-truth" set (lines 287 - 289).

4. Katdetectr does outperform other packages for high TMB samples (≧10). But those are rela-tively few (< 10% of samples). Should state this clearly in text.

We have added the number of currently-investigated WGS samples with a TMB≧ 10 (n = 20) to our manuscript (line 186).

The large pan-cancer analysis by Priestley et al. (2019)[1] on metastatic cancers revealed that 17.7% of examined malignancies reveal TMB ≧ 10 and that this is not a rare occurrence for several malignancies. In particular, metastatic skin and lung malignancies reveal 55-60% cases with such elevated TMB. We have further elaborated these potential use-cases within our discussion (line 260 - 261).

[1] Priestley P, Baber J, Lolkema MP, et al. Pan-cancer whole-genome analyses of metastatic solid tumours. Nature. 2019;575(7781):210-216. doi:10.1038/s41586-019-1689-y

5. The runtime data would be better represented by violin plots. Having many data points bunched together isn't helpful to visualize the distributions.

As per reviewer request, we have replaced the boxplots in fig. 2C and suppl. fig. 2 with violin plots and individual data-points.

6. I tested the katdetectr package and noticed something peculiar about the documentation. In section 6 "More parameter settings", there's a disclaimer that the developers did not test such settings. Doesn't seem like a good practice to put that in there if the devs themselves don't know how the function will behave.

We thank the reviewer for extensively investigating katdetectr and commenting on the accompanying vignette.

We would like to emphasize that we have thoroughly tested all available functions presented within katdetectr (incl. unit-testing) to ensure future sanity and proper function. In addition, katdetectr adheres to the BioConductor guidelines and follows their formal programmatic style, testing and documentation.

We merely wished to highlight additional functionality of the presented methodology and the flexibility of the user-available parameters by showcasing an additional use-case involving clustered mutations which do not necessarily adhere to the canonical kataegis ruleset. Whilst we ensured that these additional results were sane, we did not perform an extensive evaluation and comparison of these additional functionalities similar to those we performed for the detection of kataegis.

We agree with the reviewer that this could be misconstrued and derailing from the main functionality of katdetectr, as evaluated within this manuscript, and have removed this section from the vignette.

We updated katdetectr on BioConductor, but please note that the Bioconductor release branch is only updated twice per year (incl. the change in the vignette). The most current version of katdetectr which already includes this change is available from GitHub: https://github.com/ErasmusMC-CCBC/katdetectr

Reviewer #2: major revision

This manuscript presents a clever tool of hypermutation detection with changepoint analysis-based R languages, katdetectr. The authors have constructed the R package based on the changepoint package of Killick and Eckley.

1. In the mutation processing step, the author stated that "the imported variants are pre-processed such that, per chromosome, all variants are sorted in ascending order based on their genomic position. Overlapping variants are merged into a single record." What does "all variants" refer to?

With all variants, we referred to all the genomic variants as supplied by the user within their VCF, MAF or user-curated VRanges object. Users can perform pre-filtering of genomic variants by utilizing the VRanges object (e.g., as generated from a VCF) and supplying this VRanges into the kataegis detection method. This VRanges can house SNVs, (long) InDels and structural variants and all will be used for downstream kataegis detection if present.

We apologize for the previous omission of details and have extended this within our manuscript (line 358 - 359).

2. Are other variants, e.g., long Indel and structure variation included?

As also mentioned in the previous comment (#1), all forms of genomic variants can be supplied to katdetectr and used for subsequent kataegis detection. The presented analysis and evaluation of kataegis calls as presented within this manuscript was performed on SNVs-only as at least one package only imported SNVs.

Katdetectr merges (partially) overlapping genomic variants (regions) using IRanges::reduce() and from this generates a single record with the 5′-most shared position as reference anchor (start position), an X as reference allele, XX as alternative allele and containing information detailing which variant records were merged.

However, it would be advisable to filter all or large (e.g., >1kb) structural variations beforehand as these could potentially overlap with many (smaller) genomic variants resulting in a potential loss of kataegis detection.

We have extended our methodology with these details on merging overlapping variants (line 358 - 360)

3. How do the other tools deal with such variants, and what's your consideration for this treatment?

To better address this interesting question, we performed an investigation on how the alternative packages handle (long) and overlapping variants as the respective papers, manuscripts, vignettes and manuals lack much (if any) detail on this topic.

We added an additional script to our public repository which we used to assess the behavior of these packages regarding overlapping variants: https://github.com/ErasmusMC-CCBC/evaluation_katdetectr/blob/main/notebooks/R/checking_overlapping_variants.Rmd

With this script, we generated a small synthetic sample-set of (non-)overlapping variants:
1 InDel (1200 kb)
10 random SNVs
10 kataegis SNVs
9 SNVs that overlap with one or more of the previous
10 SNVs at exactly the same genomic location

If no merging is performed, 40 variants should be present in the resulting data-tables. If merging is performed (such as in katdetectr), only 22 variants should be present. This allows us to empirically determine the (default) behavior of the packages as the documentation and respective application notes

are scarce on details regarding overlapping variants.

Please see below, comment #4, for the details of this analysis. In summary, none of the (other) evaluated packages performed merging of overlapping variants.

Maftools
We did not find relevant parameters regarding overlapping variants and no reference to overlapping variants in the documentation, paper or manual of this tools.

From our test-set, we observed that the overlapping variants are not merged.

SeqKat
We did not find relevant parameters regarding overlapping variants and no reference to overlapping variants in the documentation, paper or manual of this tools. SeqKat furthermore only allows the import of a BED file containing SNVs, disregarding anything larger than 1bp.

From our remaining test-set of SNVs-only, we observed that the overlapping variants are not merged.

ClusteredMutations
We did not find relevant parameters regarding overlapping variants and no reference to overlapping variants in the documentation, paper or manual of this tools.

From our test-set, we observed that the overlapping variants are not merged.

SigProfilerClusters
We did not find relevant parameters regarding overlapping variants and no reference to overlapping variants in the documentation, paper or manual of this tools.

From our test-set, we observed that the overlapping variants are not merged.

Kataegis
We did not find relevant parameters regarding overlapping variants and no reference to overlapping variants in the documentation, paper or manual of this tools.

From our test-set, we observed that the overlapping variants are not merged.

4. What are "overlapping variants"?

Please see comment #3 for an explanation of the algorithm and internal handling.
Please see the supplementary rebuttal pdf file that contains mathematical expressions which we use to respond to this important question.

5. Why should they be merged?

We deemed merging overlapping genomic variants necessary as we currently do not implement phasing of alleles or include clonal cancer fractions for detection of kataegis to ease the interpretation and accessibility of katdetectr for a general audience. Therefore, if two overlapping variants would not be merged, they would contain a negative or 0 IMD. This could inflate the detection of kataegis whilst likely reflecting an admixture of clones with mutations on alternate genomes / haplotypes or an altogether complex genomic rearrangement. (line 360)

Please note that any merged records will always contain unique metadata (“revmap”) detailing the merged variants, a reference allele of X and an alternative allele of XX. This allows user to manually further investigate these regions.

6. Are there any outcomes of these treatments here?

Within all 1024 synthetic samples constituting a total of 21,299,360 SNVs, 4592 SNVs (0.02%) were merged to a single datapoint.

Within all 507 evaluated WGS samples (Alexandrov et al. 2013) constituting a total of 3,382,751 SNV, no SNVs were merged which likely reflects a pre-filtering step within the initial dataset by the authors.

7. There is a lookup table for chromosome length of UCSC hg19 (in function_performChangepointDetection.R). Does this tool also support other reference genomes of different species or different versions of human genomes? If so, how can users change this parameter?

The previous version (v1.0.0) as deposited at submission of this article indeed only (erroneously) contained a lookup table for hg19. We previously addressed this reviewer's concern in the following git issue: https://github.com/ErasmusMC-CCBC/katdetectr/issues/1

On 26-04-2023, the release branch of BioConductor was updated which includes this update (katdetectr v1.2.0). This updated version of katdetectr contains the argument "refSeq" in detectKataegis() which can be used to specify which human reference genome (by supplying "hg19" or "hg38") should be considered. Additionally, this argument can be used to supply the necessary sequence length for analysis other genomes; allowing for the analysis of additional organisms.

We have also included additional information within the vignette detailing this, please see section: "Analyzing non-standard sequences" in the vignette accompanied with the katdetectr package (v1.2.0): https://bioconductor.org/packages/devel/bioc/vignettes/katdetectr/inst/doc/General_overview.html

8. The authors tested four algorithms of changepoint package for kataegis detection, and found the PELT algorithm outperformed the others. The authors have described the results roughly, could the authors state the reasons in mathematical aspect more detailly? And are these methods recommended in another scenario?

Whilst this is an interesting question, we feel that Killick and Eckley[1,2] have already expertly detailed the various mathematical intricacies of these algorithms, as employed within the changepoint package. These excellent works contain the information concerning; mathematical proofs, computational complexity, definitions of the search algorithms, possible loss functions and their implications, methods for guarding against overfitting, changes in mean, changes in variance, changes in mean and variance, and more examples.

Within our manuscript, we opted to forego this introduction to focus on the empirical performance of these search methods in the context of kataegis detection within WGS data.

[1] R. Killick, P. Fearnhead & I. A. Eckley (2012) Optimal Detection of Changepoints With a Linear Computational Cost, Journal of the American Statistical Association, 107:500, 1590-1598, DOI: 10.1080/01621459.2012.737745

[2] Killick, R., & Eckley, I. A. (2014). changepoint: An R Package for Changepoint Analysis. Journal of Statistical Software, 58(3), 1–19. https://doi.org/10.18637/jss.v058.i03

9. I noticed you have added one pseudo IMD in the distance from the last variant to the end of the DNA

sequence to make the rates detection in change point analysis equal the mutation rate of the entire chromosome. Why this process is necessary?

Please see the supplementary rebuttal pdf file that contains mathematical expressions which we use to respond to this relevant question.

10. Except for these four algorithms, do you have any plan for implementing other algorithms for this package?

To our understanding, PELT is the current state-of-the-art search algorithm for changepoint analysis. Therefore, have currently employed this as the default algorithm. Nevertheless, we implemented katdetectr in a flexible and open-source manner which allows us or other contributors to easily implement additional search methods when requested. As PELT provided us with overall good results regarding kataegis detection, we do not foresee the immediate usage of alternate methods.

11. In the performance evaluation, you have the same variants files tested with different tools with default parameters. As we know, the tools with PCF algorithms may have parameters of penalty for each discontinuity in the curve. What are these parameters set default in these tools?

Both MafTools and Kataegis mostly employ a Piecewise Constant Fit (PCF) methodology for kataegis detection. To the best of our knowledge, we did not discern a relevant parameter in maftools (maftools::rainfallPlot()) which concerns the "penalty for each discontinuity" therefore we cannot comment on this further.

Within the package Kataegis, the kataegis::kata() function contains the "gamma" parameter for which the manual states that this sets the "penalty for each discontinuity in the curve" and is by default set to 25 (and was also left default during our performance evaluation).

We have sought to perform all alternative tools utilizing their hard-coded or otherwise suggested default settings as mentioned by the authors in their respective manuscripts and/or manuals to the best of our ability (line 600 - 618). Katdetectr was likewise performed with its defaults settings as described within our manuscript and/or hard-coded default values.

12. Are there any influences on the kataegis detection?

As also mentioned in comment #11, we have sought to perform all alternative tools utilizing their hard-coded or otherwise suggested default settings as mentioned by the authors in their respective manuscripts and/or manuals to the best of our ability (line 621 - 638). Katdetectr was likewise performed with its defaults settings as described within our manuscript and/or hard-coded default values. We have not performed additional parameter sweeps for the alternative packages as we argue that the default settings will be used by the majority of users. We therefore cannot discard that fine-tuning the parameters would have an influence on the current evaluation.

We have added this limitation to the discussion (line 307 - 312).

13. For different tools you have convert the datasets to different formats, i.e., MAF, BED, why do you choose MAF as the input format and how do you keep the input data consistency in all these different formats?

Within katdetectr, we provide functions to import VCF and MAF files or custom VRanges. However, several other evaluated packages were only capable of importing MAF or BED files. Therefore, we converted the variant data into the preferred formats as specified in the respective manuals of each package. Each time, we checked the consistency of the transformed data to exclude possible artefacts during conversion.

All utilized code for the importing and transformation of the data can be found in our GitHub repository:https://github.com/ErasmusMC-CCBC/evaluation_katdetectr/

14. For the evaluation scores, could the authors provide raw score of true positive and true negative other than TPR and TNR?

Supplementary tables 1 and 2 contain the raw data detailing all true positives, false positives, true negatives, and false negatives per package for the synthetic and WGS datasets respectively.

15. In addition, the deposited data for performance evaluation is not accessible outside my workplace. And more detailed instructions are necessary for the data. After I loaded the data named parameters_synthetic_data.RData in R, I was lost for deeper looking into the data. When I tried to direct the loaded data to an object, a text of "chr "parameters"" was echoed.

To ease further reproducibility of our work, we have implemented a Jupyter (R) Notebook in which the various steps of the comparison can be reproduced in a virtual environment (or within a local R environment when installing the IRkernel package): https://github.com/ErasmusMC-CCBC/evaluation_katdetectr/blob/main/notebooks/1.EvaluatePackages.ipynb

In addition, this notebook contains a code snippet (using zen4R) which can automatically download all our initial input and generated results directly from Zenodo:https://dx.doi.org/10.5281/zenodo.6810477

These downloaded data can then be used in the downstream visualization and performance evaluations code-blocks. We hope this eases the reviewer reproduction of the initial dataset and following steps leading to the (re-)production of all presented figures and tables.
