# Supplementary material for: Katdetectr: an R/bioconductor package utilizing unsupervised changepoint analysis for robust kataegis detection
Source: Gigascience. 2023 Oct 17;12:giad081. doi: 10.1093/gigascience/giad081 (PMC10580377; doi:10.1093/gigascience/giad081)
Supplement: giad081_Reviewer_1_Report_Original_Submission — Kin Chan -- 4/3/2023 Reviewed [file giad081_reviewer_1_report_original_submission.pdf]

Reviewer Report

Title: Katdetectr: An R/Bioconductor package utilizing unsupervised changepoint analysis for robust kataegis detection

Version: Original Submission **Date:** 4/3/2023

Reviewer name: Kin Chan

Reviewer Comments to Author:

Overview: In this short manuscript, Hazelaar et al. describe a new software package written in R, called "katdetectr". This package can be useful as an addition to existing computational tools for identifying and characterizing kataegis in cancer genomes. The paper then compares katdetectr favorably against other software for detecting kataegis, using synthetic and real cancer data. Overall, the paper is fine and the katdetectr package is a nice addition for researchers' toolbox. I would suggest that the authors make the following improvements.1. Choose a convention for decimal point and digit separator, then stick with it. "." was used as both the decimal point and digit separator for large numbers, which gets confusing. Typically, "." Is used for decimal point and "," is used for digit separator.2. The Introduction is so abbreviated that it doesn't serve much purpose. Either flesh it out with more information or just drop it completely. This journal accepts papers that go right into results, so it's fine. But the authors should also consider if writing a more expansive introduction can make the paper more accessible to readers who aren't already as knowledgeable.3. The biggest issue is using the 2013 Alexandrov kataegis calls as "ground truth" when multiple packages published since then detect 102 loci that Alexandrov (2013) missed. Seems like it would be much more sensible to use the calls from the 2020 PCAWG paper instead: https://doi.org/10.1038/s41586-020-1969-6. The data are controlled access, but it should be possible to get them.4. Katdetectr does outperform other packages for high TMB samples (â‰§10). But those are relatively few (< 10% of samples). Should state this clearly in text.5. The runtime data would be better represented by violin plots. Having many data points bunched together isn't helpful to visualize the distributions.6. I tested the katdetectr package and noticed something peculiar about the documentation. In section 6 "More parameter settings", there's a disclaimer that the developers did not test such settings. Doesn't seem like a good practice to put that in there if the devs themselves don't know how the function will behave.

Level of Interest

Please indicate how interesting you found the manuscript: Choose an item.

Quality of Written English

Please indicate the quality of language in the manuscript: Choose an item.

Declaration of Competing Interests

Please complete a declaration of competing interests, considering the following questions:

- Have you in the past five years received reimbursements, fees, funding, or salary from an organisation that may in any way gain or lose financially from the publication of this manuscript, either now or in the future?
- Do you hold any stocks or shares in an organisation that may in any way gain or lose financially from the publication of this manuscript, either now or in the future?
- Do you hold or are you currently applying for any patents relating to the content of the manuscript?
- Have you received reimbursements, fees, funding, or salary from an organization that holds or has applied for patents relating to the content of the manuscript?
- Do you have any other financial competing interests?
- Do you have any non-financial competing interests in relation to this paper?

If you can answer no to all of the above, write 'I declare that I have no competing interests' below. If your reply is yes to any, please give details below.

I declare that I have no competing interests

I agree to the open peer review policy of the journal. I understand that my name will be included on my report to the authors and, if the manuscript is accepted for publication, my named report including any attachments I upload will be posted on the website along with the authors' responses. I agree for my report to be made available under an Open Access Creative Commons CC-BY license (http://creativecommons.org/licenses/by/4.0/). I understand that any comments which I do not wish to be included in my named report can be included as confidential comments to the editors, which will not be published.

Choose an item.

To further support our reviewers, we have joined with Publons, where you can gain additional credit to further highlight your hard work (see: https://publons.com/journal/530/gigascience). On publication of this paper, your review will be automatically added to Publons, you can then choose whether or not to claim your Publons credit. I understand this statement.

Yes Choose an item.
