# Supplementary material for: Katdetectr: an R/bioconductor package utilizing unsupervised changepoint analysis for robust kataegis detection
Source: Gigascience. 2023 Oct 17;12:giad081. doi: 10.1093/gigascience/giad081 (PMC10580377; doi:10.1093/gigascience/giad081)
Supplement: giad081_Reviewer_2_Report_Original_Submission — Jian Li -- 4/16/2023 Reviewed [file giad081_reviewer_2_report_original_submission.pdf]

Reviewer Report

Title: Katdetectr: An R/Bioconductor package utilizing unsupervised changepoint analysis for robust kataegis detection

Version: Original Submission **Date:** 4/16/2023

Reviewer name: Jian Li

Reviewer Comments to Author:

This manuscript presents a clever tool of hypermutation detection with change point analysis based R languages, katdetectr. The authors have constructed the R package based on the chagnepoint pacakge of Killick and Eckley.In the mutation processing step, the author stated that "the imported variants are pre-processed such that, per chromosome, all variants are sorted inascending order based on their genomic position. Overlapping variants are merged into a single record." What does "all variants" refer to? Are other variants, e.g., long Indel and structure variation included? How do the other tools deal with such variants, and what's your consideration for this treatment? What are "overlapping variants"? Why should they be merged? Are there any outcomes of these treatments here? There is a lookup table for choromsome length of UCSC hg19 (in function_performChangepointDetection.R). Does this tool also support other reference genomes of different specisis or different versions of human genomes? If so, how can users change this parameter?The authors tested four algorithms of changepoint package for kataegis detection, and foind the PELT algorithm outperformed the others. The authors have descrived the results roughly, could the authors stated the reasons in mathematical aspect more detailly? And are these methods recommended in other senario? I noticed you have added one pseudo IMD in the distance from the last variant to the end of the DNA sequence to make the rates detection in change point analysis equal the matation rate of the entire chromosome. Why this process is neccessary? Except for these four algorithms, do you have any plan for implementing other algorithms for this packages?In the performace evalutation, you have the same variants files tested with different tools with default parameters. As we know, the tools with PCF algorithms may have parameters of penalty for each discontinuity in the curve. What are these parametered set defaultly in these tools? Are there any influences on the kataegis detection? For different tools you have convert the datasets to different formats, i.e., MAF, BED, why do you choose MAF as the input format and how do you keep the input data consistensy in all these different formats? For the evaluation scores, could the authors provied raw score of true positive and true negative other than TPR and TNR? In addition, the deposited data for performace evalutation is not accessible outside my workplace. And more detailed instructions are neccesary for the data. After I loaded the data named parameters_synthetic_data.RData in R, I was lost for deeper looking into the data. When I tried to direct the loaded data to an object, a text of "chr "parameters"" was echoed.

Level of Interest

Please indicate how interesting you found the manuscript: Choose an item.

Quality of Written English

Please indicate the quality of language in the manuscript: Choose an item.

Declaration of Competing Interests

Please complete a declaration of competing interests, considering the following questions:

- Have you in the past five years received reimbursements, fees, funding, or salary from an organisation that may in any way gain or lose financially from the publication of this manuscript, either now or in the future?
- Do you hold any stocks or shares in an organisation that may in any way gain or lose financially from the publication of this manuscript, either now or in the future?
- Do you hold or are you currently applying for any patents relating to the content of the manuscript?
- Have you received reimbursements, fees, funding, or salary from an organization that holds or has applied for patents relating to the content of the manuscript?
- Do you have any other financial competing interests?
- Do you have any non-financial competing interests in relation to this paper?

If you can answer no to all of the above, write 'I declare that I have no competing interests' below. If your reply is yes to any, please give details below.

I declare that I have no competing interests

I agree to the open peer review policy of the journal. I understand that my name will be included on my report to the authors and, if the manuscript is accepted for publication, my named report including any attachments I upload will be posted on the website along with the authors' responses. I agree for my report to be made available under an Open Access Creative Commons CC-BY license (http://creativecommons.org/licenses/by/4.0/). I understand that any comments which I do not wish to be included in my named report can be included as confidential comments to the editors, which will not be published.

Choose an item.

To further support our reviewers, we have joined with Publons, where you can gain additional credit to further highlight your hard work (see: https://publons.com/journal/530/gigascience). On publication of this paper, your review will be automatically added to Publons, you can then choose whether or not to claim your Publons credit. I understand this statement.

Yes Choose an item.
